# Molecular Basis of Essentiality of Early Critical Steps in the Lipopolysaccharide Biogenesis in *Escherichia coli* K-12: Requirement of MsbA, Cardiolipin, LpxL, LpxM and GcvB

**DOI:** 10.3390/ijms22105099

**Published:** 2021-05-12

**Authors:** Patrycja Gorzelak, Gracjana Klein, Satish Raina

**Affiliations:** Unit of Bacterial Genetics, Gdansk University of Technology, 80-233 Gdansk, Poland; patrycja.gorzelak@gmail.com

**Keywords:** LPS, Kdo transferase, lauroyl acyltransferase LpxL, myristoyl transferase LpxM, heptosyltransferase, LapC (YejM), LpxC, cardiolipin, MsbA, GcvB

## Abstract

To identify the physiological factors that limit the growth of *Escherichia coli* K-12 strains synthesizing minimal lipopolysaccharide (LPS), we describe the first construction of strains devoid of the entire *waa* locus and concomitantly lacking all three acyltransferases (LpxL/LpxM/LpxP), synthesizing minimal lipid IV_A_ derivatives with a restricted ability to grow at around 21 °C. Suppressors restoring growth up to 37 °C of Δ(*gmhD-waaA*) identified two independent single-amino-acid substitutions—P50S and R310S—in the LPS flippase MsbA. Interestingly, the cardiolipin synthase-encoding gene *clsA* was found to be essential for the growth of Δ*lpxLMP*, Δ*lpxL*, Δ*waaA*, and Δ(*gmhD-waaA*) bacteria, with a conditional lethal phenotype of Δ(*clsA lpxM*), which could be overcome by suppressor mutations in MsbA. Suppressor mutations *basS* A20D or *basR* G53V, causing a constitutive incorporation of phosphoethanolamine (*P-EtN*) in the lipid A, could abolish the Ca^++^ sensitivity of Δ(*waaC eptB*), thereby compensating for *P-EtN* absence on the second Kdo. A single-amino-acid OppA S273G substitution is shown to overcome the synthetic lethality of Δ(*waaC surA*) bacteria, consistent with the chaperone-like function of the OppA oligopeptide-binding protein. Furthermore, overexpression of GcvB sRNA was found to repress the accumulation of LpxC and suppress the lethality of LapAB absence. Thus, this study identifies new and limiting factors in regulating LPS biosynthesis.

## 1. Introduction

The defining and most conserved feature of Gram-negative bacteria is the presence of an asymmetric outer membrane (OM), which is essential for their viability [1]. Lipopolysaccharide (LPS) constitutes the major component of the OM, and is one of the main virulence factors of pathogenic Gram-negative bacteria [1,2,3]. The chemical composition of LPS endows it with properties that provide the permeability barrier function, thereby preventing the entry of bulky hydrophobic and toxic compounds into the bacterial cell [1]. LPS is a complex glycolipid comprised of a hydrophobic membrane-anchored lipid A and a core oligosaccharide, which is linked to the *O*-antigen in smooth-type bacteria [2]. The lipid A part constitutes the principal endotoxin, and consists of an acylated and 1,4′ bisphosphorylated β(1→6)-linked glucosamine (GlcN) disaccharide [2,3,4]. In *Escherichia coli*, the core oligosaccharide can be subdivided into the inner and outer cores. The inner core generally contains a conserved structural element of 3-deoxy-α-D-*manno*-oct-2-ulopyranosonic acid (Kdo), L-*glycero*-α-D-*manno*-heptopyranose (Hep), and phosphate residues [5].

Lipid A biosynthesis begins with the LpxA-catalyzed acylation of UDP-GlcNAc [2,3,4,6,7]. This product is further deacylated by the Zn^++^-dependent deacylase LpxC, whose quantities are tightly regulated by LapB, LapC, FtsH, and HslUV, constituting the first committed step in LPS biosynthesis, as the first reaction catalyzed by LpxA is energetically unfavorable [7,8,9,10,11,12,13]. Following the deacylation step, four additional enzymes (LpxD, LpxH, LpxB, and LpxK) act sequentially to generate the lipid IV_A_ precursor [2,4,14,15,16]. This lipid IV_A_ then serves as an acceptor for the Kdo transferase WaaA. In *E. coli* K-12, WaaA transfers two Kdo residues from CMP-Kdo, generating an α(2→4)-linked Kdo disaccharide attached α(2→6) to the non-reducing GlcN residue of lipid IV_A_ [17]. Up to this step, all seven required enzymes are essential for bacterial viability [3]. The product of this reaction results in the synthesis of Kdo_2_-lipid IV_A_, which further acts as an essential intermediate in LPS synthesis. The incorporation of Kdo residues ensures late acylation steps to generate hexaacylated lipid A (Kdo_2_-LA_hexa_), and further extension via the incorporation of various sugars by different glycosyltransferases to produce complete LPS [2,4,5,16].

**Figure 1:**
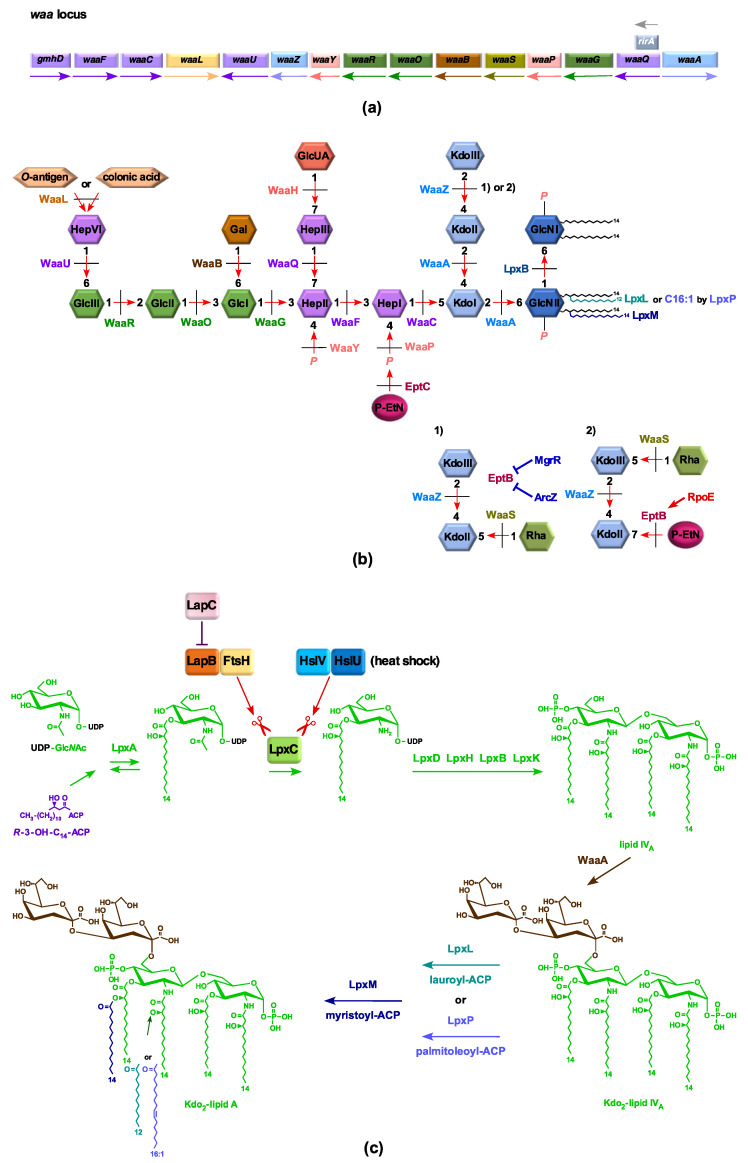
LPS biosynthetic genes and their structural modifications: (**a**) Schematic drawing of the *waa* locus. Orientations of three operons and genes encoding various biosynthetic enzymes are shown. Also shown is co-transcription of RirA sRNA with *waaQ*. (**b**) Depiction of the role of various glycosyltransferases and acyltransferases in LPS biosynthesis. The bottom panel 1 highlights inner core alterations that result in the incorporation of a third Kdo and Rha. In panel 2, a shift of Rha attachment to the third Kdo upon the EptB-dependent incorporation of phosphoethanolamine on the second Kdo, when RpoE is induced, is shown. (**c**) Schematic drawing of the Raetz pathway of biosynthesis of Kdo_2_-LA_hexa_. Critical steps that regulate LpxC levels are depicted.

*E. coli* uses three acyltransferases—LpxL, LpxM, and LpxP—to incorporate late acyl chains, usually after the incorporation of Kdo on lipid IV_A_ to generate hexaacylated lipid A, using acyl-carrier protein-activated fatty acids as co-substrates [18,19]. Under ambient growth conditions, a lauroyl chain is first transferred by LpxL to the OH group of the amide-bound (*R*)-3-hydroxymyristate at position 2′ [20]. However, this step can be replaced up to 80% by the incorporation of palmitoleate by LpxP at low temperatures, whose transcription is upregulated upon the RpoE induction [19,21]. These pentaacylated lipid IV_A_ derivatives serve as acceptors for LpxM-mediated myristolyation at position 3′ to produce hexaacylated lipid A [22]. In *E. coli* K-12, the *lpxL*, *lpxP*, and *lpxM* genes are not individually essential for viability, although Δ*lpxL* mutants exhibit a temperature-sensitive phenotype in rich media above 33 °C [18,19,23]. The importance of late acyltransferases is reflected by the fact that a triple deletion derivative, lacking all three late acyltransferases, is viable only at 30 °C or below in minimal media [18,19]. This can be explained due to lipid IV_A_ being a poor substrate for the LPS flippase MsbA, compared to hexaacylated lipid A [18,24,25]. Interestingly, Δ(*lpxL lpxM lpxP*) strains with an intact *waa* locus, under growth conditions that promote the incorporation of non-stoichiometric modifications, synthesize LPS nearly exclusively composed of a glycoform with a third Kdo+Rhamnose (Rha) [19]. Although WaaA is essential under standard laboratory growth conditions, we previously reported the construction of suppressor-free strains lacking the *waaA* gene at 21 °C [19]. Such strains were shown to synthesize glycosylation-free LPS composed of lipid IV_A_ derivatives [19]. Importantly, under slow-growth conditions (at 21 °C), where the growth rate of bacteria is significantly reduced, Δ*waaA* strains were shown to incorporate lauroyl, palmitoyl, myristoyl, and palmitoleate acyl chains, generating a mixture of pentaacylated and hexaacylated lipid IV_A_ species even in the absence of Kdo transferase [19]. Analysis of glycerophospholipids and lipid IV_A_ of suppressor-free Δ*waaA* strains also revealed that they contain a significant amount of phospholipids—particularly cardiolipin, which is usually a minor component of phospholipids [19]. However, two independent groups have also reported the construction of glycosylation-free LPS derivatives, either in the presence of extra doses of LPS ABC transporter MsbA, or when genes encoding late acyltransferases were overexpressed, or in the presence of extragenic suppressor mutations [25,26]. Moreover, strains devoid of the entire *waa* locus (Figure 1) that lack WaaA as well as WaaZ Kdo transferases have not been reported. Furthermore, Δ(*lpxL lpxM lpxP*) derivatives without the *waa* locus have not been described thus far.

Biosynthesis of LPS, phospholipids, and peptidoglycan are intricately linked. LPS and phospholipids share (*R*)-3-hydroxymyristate as a common metabolic precursor, and a tight balance is maintained in the quantities of these two essential components of the cell envelope [10,11]. This is achieved through the regulation of LpxC quantity and FabZ activity [19,27]. LpxC quantity is controlled by the proteolytic activity of the FtsH/LapB/LapC complex and HslUV proteases [12,13]. FtsH proteolytic activity for LpxC requires the heat shock protein LapB, whereas LapC acts antagonistically to prevent excessive degradation of LpxC, and thus together maintain a balanced biosynthesis of LPS [9,12,13]. Similarly, UDP-GlcNAc serves as a precursor for LPS as well as peptidoglycan synthesis [3]. Thus, it is not surprising that suppressors of deletion derivatives of the *lapB* gene map to genes whose products are involved in all three essential components of cell envelope biosynthesis, and any perturbance in any of these components triggers the cell envelope stress response under the control of the RpoE sigma factor [9].

Lipid A and the inner core are more conserved in bacteria, although they often carry non-stoichiometric modifications, some of which are incorporated upon challenge to different stresses, like changes in pH, concentrations of specific ions—such as Mg^++^, Ca^++^, Fe^+++^, or Zn^++^—and phosphate starvation [19,28]. Among the non-stoichiometric substitutions commonly observed in the lipid A are the incorporation of phosphoethanolamine (*P-EtN*) and 4-amino-4-deoxy-L-arabinose (L-Ara4N), which are known to confer resistance to cationic antimicrobial peptides, such as polymyxin B [28]. Interestingly, strains lacking either WaaC or WaaF heptosyltransferases preferentially incorporate *P-EtN* on the second Kdo at the expense of *P-EtN* in the lipid A part due to the RpoE-dependent transcriptional upregulation of the *eptB* gene in such genetic backgrounds [19,29]. However, up to now, the physiological significance of preferential *P-EtN* incorporation on the second Kdo in Δ*waaC* or Δ*waaF* backgrounds is not known, although Δ(*waaC eptB*) mutants exhibit sensitivity to sub-lethal concentrations of Ca^++^ [19,29,30,31,32,33]. Moreover, under RpoE-inducing conditions, strains with an intact *waa* locus synthesize LPS with the lipid A linked to a branched tetra-saccharide containing a third Kdo+Rha, with *P-EtN* on the second Kdo and a concomitant truncation of the terminal Hexose–Heptose, reflecting another important role of EptB function and the study of the function of the entire *waa* locus (Figure 1) [33].

Apart from the essential *waaA* gene, all other genes in the entire *waa* locus are dispensable, although truncations in the inner core cause permeability defects and the induction of envelope stress response [5]. Additionally, the removal of genes whose products are involved in the early steps of LPS inner core biosynthesis (*waaC*, *waaF*, *waaG*, *waaQ*, and *waaP*) confers a growth disadvantage at high temperatures, and causes defects in bacterial motility [3,33,34]. The importance of WaaC in *E. coli*’s physiology is further manifested by the synthetic lethality exhibited when the periplasmic folding factor SurA is also absent, presumably due to hyperinduction of the RpoE-dependent stress response and outer membrane defects [19]. Furthermore, the removal of the *waaZ* gene encoding the Kdo transferase responsible for the attachment of a third Kdo confers a synthetic growth defect in the Δ(*lpxL lpxM lpxP*) background [33].

In this study, we describe the construction of strains devoid of all genes, whose products are involved in LPS core biosynthesis, including WaaA and WaaZ Kdo transferases. Such strains are shown to synthesize the glycosylation-free lipid IV_A_ precursor with the restricted ability to grow under slow-growth conditions (21 °C) without providing any extra copy of MsbA. However, suppressors mapping to the *msbA* gene with single-amino-acid alterations in the lipid A-binding region were identified, which allowed their growth up to 37 °C in rich media. As cardiolipin species were observed in Δ*waaA* and Δ(*lpxMP*) derivatives, we also investigated the requirement of the *clsA* gene encoding the major cardiolipin synthase [35]. We further addressed the molecular basis of sensitivity to Ca^++^ of Δ(*waaC eptB*) mutants by isolating Ca^++^-resistant suppressors and characterizing their LPS. We show that such suppressors contain a single-amino-acid substitution either in the *basS* or the *basR* gene, leading to *P-EtN* and L-Ara4N incorporation in the lipid A. Furthermore, a suppressor mutation in the *oppA* gene encoding a periplasmic oligopeptide-binding protein was found to rescue the synthetic lethality of Δ(*waaC surA*) mutants. In further investigation of LpxC and LapA/B function in the regulation of LPS synthesis, we show that overexpression of GcvB regulatory RNA represses LpxC synthesis. GcvB is one of the most conserved sRNAs, is thought to regulate nearly 1–2% of mRNAs in *E. coli* and *Salmonella*, and its regulon members include genes whose products are involved in the transport of short peptides/amino acids and/or act as transcription factors, such as CsgD, Lrp, and PhoP [36,37,38,39,40]. However, its role in regulating any LPS biosynthetic gene has not been reported. Thus, this study highlights the importance of early steps in LPS biosynthesis and novel players in maintaining cell envelope integrity.

## 2. Results

### 2.1. Strains Lacking the Entire waa Locus Synthesize LPS Composed of Glycosylation-Free Lipid IV_A_ and Can Incorporate Lauryl, Myristoyl, and Palmitoleate Chains under Slow-Growth Conditions

Previously, we described panels of suppressor-free strains that synthesize LPS containing only Kdo_2_-lipid IV_A_ or only lipid IV_A_ species. The growth of such strains was found to be restricted to minimal media in the temperature range of 21–23 °C, consistent with the known reduced recognition of lipid IV_A_ as a substrate of MsbA flippase compared to hexaacylated lipid A [19,41]. In *E. coli* K-12, most of the genes whose products are involved in LPS core biosynthesis, including various glycosyl/heptosyltransferases and sugar nucleotide kinases, are organized as three operons in the *waa* locus, spanning from *gmhD* (*rfaD/htrM*) to *waaA* (Figure 1). Construction of strains that lack all glycosyltransferases is of interest in providing glycosylation-free LPS and endotoxin-negative strains in glycoengineering, and understanding factors that limit their growth. As WaaA is essential under laboratory growth conditions, deletion derivatives spanning the *waaF*–*waaA* and *gmhD*–*waaA* regions encompassing the whole *waa* locus, comprised of 14 and 15 genes, respectively, were constructed by recombineering using growth conditions of minimal media at 21 °C. To verify their suppressor-free genotype, Δ(*waaF*–*waaA*) and Δ(*gmhD*–*waaA*) mutations were transduced by bacteriophage T4-mediated transductions in standard *E. coli* K-12 laboratory strains, resulting in SR9900 and its derivatives (Table 1 and Table 2).

**Figure 2:**
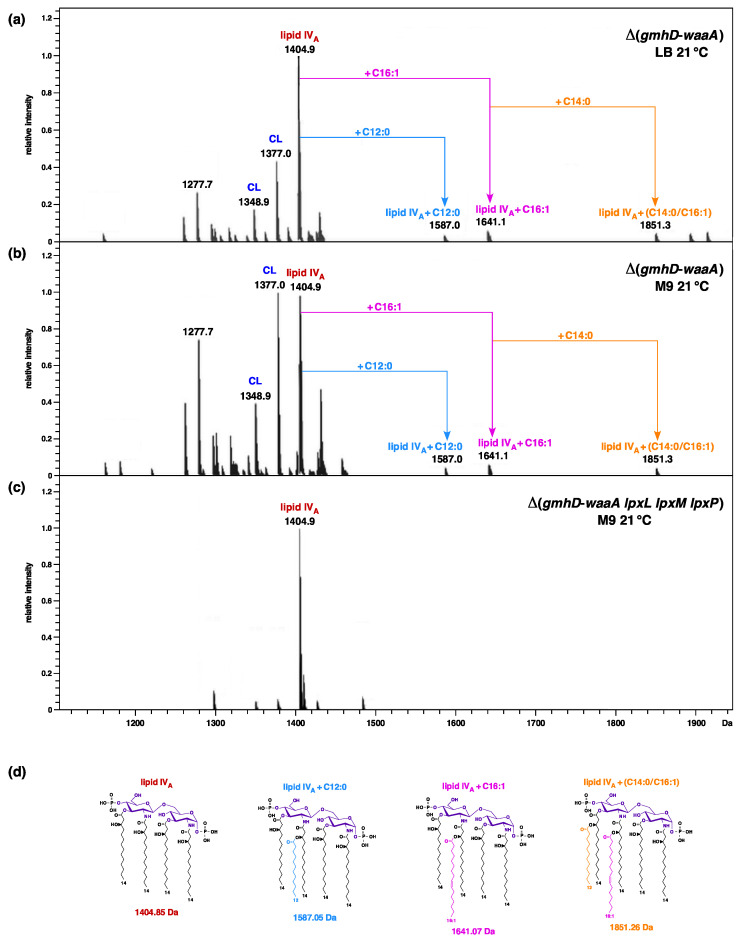
Charge deconvoluted ESI FT–ICR mass spectra in the negative ion mode of deletion derivatives of the whole *waa* locus, including WaaA Kdo transferase: (**a**) Mass spectra of LPS obtained from the Δ(*gmhD–waaA*) strain grown in LB medium at 21 °C and (**b**) in M9 minimal medium. (**c**) Mass spectra of LPS obtained from the Δ(*gmhD–waaA lpxL lpxM lpxP*) derivative grown in M9 medium at 21 °C. Mass peaks corresponding to the addition of lauroyl, myristoyl, and palmitoleate to the lipid IV_A_ species are indicated. Mass numbers are monoisotopic masses of main peaks. Mass peaks corresponding to substitutions by phosphate or sodium adducts are not labeled. Molecular ions at 1348.9 u in panels (**a**,**b**) corresponding to cardiolipin and the ion at 1377 Da can either arise due to variation in acyl chain length, or can be a cardiolipin. (**d**) The chemical structure of various tetraacylated lipid IV_A_ derivatives with predicted mass numbers.

To validate the lack of Kdo and further glycosylation, LPS was extracted from a Δ(*gmhD*–*waaA*) strain grown at 21 °C in M9 medium, which are permissive growth conditions for such a strain, and was analyzed by mass spectrometry. To analyze the LPS composition in LB medium, cultures grown in M9 minimal medium were shifted to LB medium for 6 to 8 h at 21 °C. Examination of their charge-deconvoluted spectra revealed the presence of an ion peak at 1404.8 Da, corresponding to the structure of tetraacylated 1,4′ bisphosphorylated lipid IV_A_ precursor (Figure 2a,b). Furthermore, additional mass peaks that correspond to pentaacylated and hexaacylated derivatives were also conspicuously observed, whether LPS was extracted from strains grown in either M9 or LB media, at 21 °C. Thus, the mass peak at 1641.1 Da corresponds to the predicted incorporation of the C16:1 secondary palmitoleate, which requires the activity of cold-inducible LpxP acyltransferase. Moreover, in these mass spectra, the mass peak at 1587.0 Da represents the incorporation of the secondary laurate acyl chain (Figure 2a,b). Interestingly, a mass peak at 1851.3 Da indicates an additional incorporation of the myristoyl chain, leading to the conversion of lipid IV_A_ precursor to the lipid IV_A_ C16:1+C14:0 derivative. These spectra also show the ion peaks corresponding to phospholipids that include cardiolipin species (mass peaks at 1348, 1376, and 1404 Da) (see below for details). These results confirm that under slow-growth conditions, particularly at low temperatures, lipid IV_A_ can serve as a precursor for the incorporation of secondary laurate, palmitoleate, and myristate groups, without a prior incorporation of Kdo residues, and without any additional copies of the MsbA LPS transporter.

### 2.2. Construction and Properties of Strains Lacking Three Late Acyltransferases in Δ(gmhD–waaA) Derivatives

In the above experiments, and in our previous studies describing the LPS composition of a suppressor-free Δ*waaA* strain, we showed that lipid IV_A_ can serve as an acceptor for the incorporation of secondary acyl lauroyl, myristoyl, and palmitoleate chains under slow-growth conditions when bacteria are grown at 21 °C, without the prior incorporation of Kdo residues [19]. These secondary acyl chain transferases are encoded by three distinct genes: *lpxL*, *lpxM*, and *lpxP*, respectively. It is known that at low temperatures LpxP can transfer palmitoleate instead of lauroyl residue to the OH group of the amide-bound (*R*)-3-hydroxymyristate residue at position 2′. To validate the Kdo-independent late acylation of lipid IV_A_ precursors, Δ(*waaA lpxL lpxM lpxP*) and Δ(*gmhD–waaA lpxL lpxM lpxP*) strains simultaneously lacking all three late acyltransferases and the Kdo transferase WaaA, alone or with the entire *waa* locus deleted, were constructed (Table 2). To achieve this goal, a previously well-characterized suppressor-free Δ(*lpxL lpxM lpxP*) strain, SR7781, was transformed with either a vector alone or with a plasmid expressing the *waaA* gene under the permissive growth conditions of M9 medium at 21 °C, and was used as a recipient to transduce a Δ*waaA* mutation at 21 °C in M9 minimal medium and in LB medium (Table 2). Next, in a similar transduction experiment, a Δ(*gmhD-waaA*) deletion was introduced via bacteriophage-mediated transduction. Similar numbers of transductants were obtained in such experiments at 21 °C in M9 minimal medium, whether a Δ*waaA* or a Δ(*gmhD–waaA*) strain was used as the recipient. However, no viable transductions were obtained in LB medium, even at 21 °C (Table 2).

For the LPS analysis, cultures were grown at 21 °C in M9 minimal medium. Mass spectrometric analysis of LPS/glycerophospholipid obtained from the Δ(*gmhD–waaA lpxL lpxM lpxP*) strain revealed the main mass peak at 1404.9 Da, which corresponds to a tetraacylated 1,4′ bisphosphorylated lipid IV_A_ precursor (Figure 2c). As compared to the parental Δ(*gmhD–waaA)* strain, no mass peaks corresponding to the presence of penta- or hexaacylated lipid IV_A_ derivatives were present in the spectra of LPS obtained from the Δ(*gmhD–waaA lpxL lpxM lpxP*) strain. Similar spectra showing the absence of penta- or hexaacylated derivatives in LPS Δ(*waaA lpxL lpxM lpxP*) were obtained, and hence only data from Δ(*gmhD–waaA lpxL lpxM lpxP*) are presented (Figure 2). These results further confirm that late acylation of lipid IV_A_ due to the activity of LpxL, LpxP, and LpxM can occur in Δ*waaA* derivatives at low temperatures, even without Kdo incorporation—but not when genes encoding such late acyltransferases are absent. Thus, these results establish that lipid IV_A_ can act as an acceptor for acylation by lauroyl, palmitoleate, or myristoyl transferases under slow-growth conditions of 21 °C, without the prior incorporation of Kdo, and does not require any extragenic suppressors. Additionally, incorporation of lauroyl, palmitoleate, or myristoyl chains in Δ*waaA* derivatives is dispensable for their growth in M9 minimal medium at 21 °C.

### 2.3. Isolation of Extragenic Suppressors That Allow the Growth of Either waaA or (gmhD–waaA) Deletion Derivatives at 37 °C

**Figure 3:**
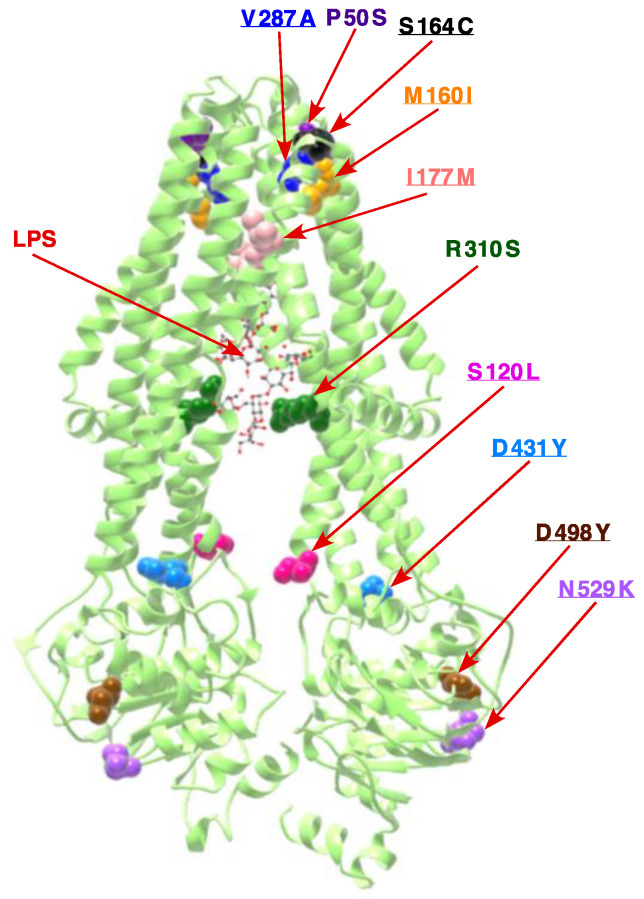
Positions of various single-amino-acid substitutions in the structure of MsbA (PDB 6BPL) [44]. Note the location of R310 in the TM6, which is lined with positively charged and polar residues, and could interact with negatively charged groups in the LPS. The position of the LPS is shown by the arrow. P50 residue is located in the putative lipid-A-binding site on the outer surface of MsbA, and could be in the pathway of lipid A release. The positions of the remaining eight single-amino-acid substitutions that rescue the conditional lethality of Δ(*lpxM clsA*) are underlined.

As described above, strains lacking the Kdo transferase can be constructed at low temperatures. However, without any suppressors, such strains exhibit a narrow growth range limited to 21–23 °C. Thus, we sought extragenic suppressors by plating several independent cultures of strains SR9900 Δ(*waaF–waaA*) and SR9902 Δ(*gmhD–waaA*) in LA medium at 33 °C. Such LA-resistant spontaneous suppressors could be obtained at a frequency of 10^−9^ after 48 h incubation. Suppressor mutations were marked with Tn*10* and used to transduce back with the linked Tn*10* insertion in Δ*waaA*, Δ(*gmhD–waaA*), and Δ(*waaF–waaA*) backgrounds, in order to ascertain the restoration of growth at 33 or 37 °C. Five Tn*10*-linked mutations were found to restore the growth of *waaA* deletion derivatives up to 37 °C in LA medium. For genetic analysis, the Tn*10*-linked mutation was moved into the wild-type background and used to recombine the Tn*10* insertion in a cosmid library covering all chromosomal genes. Further subcloning and sequencing of DNA regions adjacent to the Tn*10* insertion revealed that the Tn*10* insertion in all of suppressors was linked to the *msbA* gene. Next, the chromosomal DNA of five thusly obtained LA-resistant *waaA*-suppressor-containing strains that restore growth up to 37 °C was used to PCR-amplify the *msbA* and downstream *lpxK* genes using appropriate oligonucleotides, and then subjected to DNA sequence analysis. This DNA sequence revealed two strains to contain a single-amino-acid alteration leading to an exchange of Pro50 to Ser (CCA to TCA), while three other strains contained an exchange of Arg310 to Ser (CGC to AGC). The Pro50 residue is located on the TM1 of MsbA, constituting the periplasmic surface groove that is predicted to enter contact with the lipid A (Figure 3). The amino acid residue Arg310 is located in the TM6, which is rich in positively charged and polar residues. TM4 and TM6 constitute two portals on opposite sites, wherein positively charged residues such as Arg310 could interact with phosphorylated glucosamine groups in the lipid A part of the LPS, and are a part of the structure that provides carbon chain ruler properties to MsbA [44,45,46]. Thus, the identification of R310S as a suppressor of strains synthesizing only lipid IV_A_ LPS provides a sound rationale for such an MsbA variant to transport tetraacylated lipid IV_A_ with better efficiency.

### 2.4. Incorporation of P-EtN and Palmitate Chains in Δ(gmhD–waaA) with a Single-Amino-Acid Suppressor Mutation in the msbA Gene

**Figure 4:**
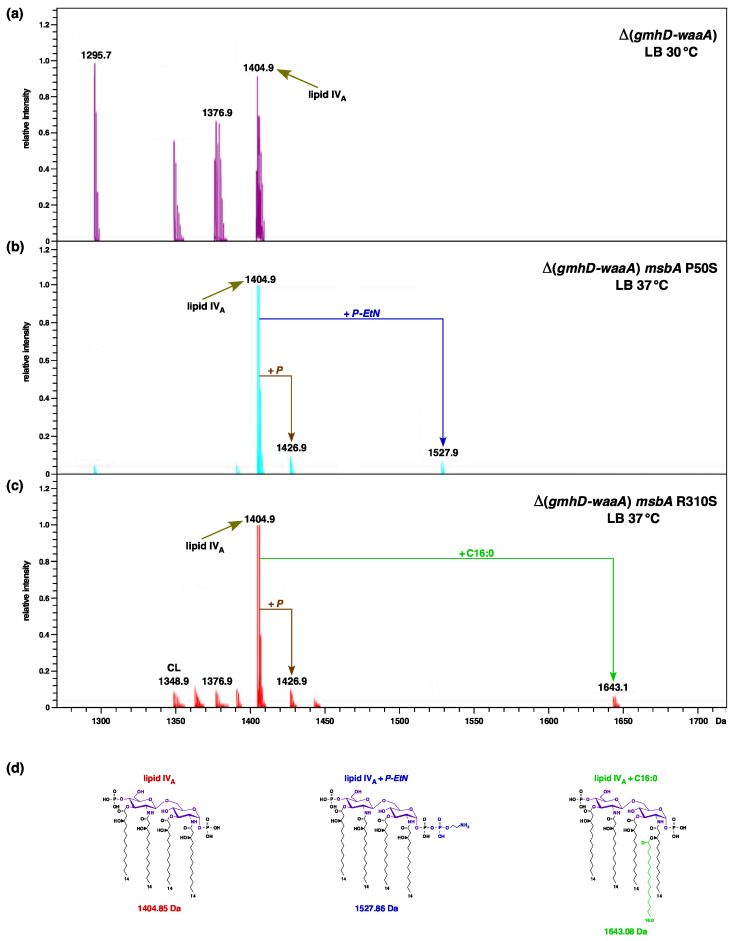
Suppressors in MsbA that restore growth up to 37 °C incorporate *P-EtN* and palmitoyl modifications in the lipid IV_A_ precursor that occur after translocation: (**a**) Mass spectra of LPS obtained from the Δ(*gmhD–waaA*) strain grown at 30 °C for 6 h after a shift from permissive growth conditions of 21 °C. (**b**) Mass spectra of LPS obtained from the Δ(*gmhD–waaA*) *msbA* P50S strain grown at 37 °C in LB medium. (**c**) Mass spectra of LPS obtained from the Δ(*gmhD–waaA*) *msbA* R310S strain grown at 37 °C in LB medium. Charge deconvoluted ESI FT–ICR mass spectra in negative ion mode are presented. The mass numbers refer to monoisotopic peaks with the proposed composition. Note the addition of *P-EtN* and C16:0 to the lipid IV_A_ precursor, when MsbA suppressor mutation is present. (**d**) The chemical structure of various tetraacylated lipid IV_A_ derivatives identified in these spectra with predicted mass numbers are shown. The mass peak marked CL corresponds to the presence of cardiolipin.

It is known that lipid A modifications occur after its translocation and flipping by MsbA across the inner membrane on the periplasmic side. Furthermore, the addition of palmitate chains occurs when LPS is present in the outer membrane, due to the presence of PagP enzymes in the OM. These modifications by *P-EtN* and the addition of palmitate chains thus serve as good indicators of LPS translocation [19,25]. As tetraacylated lipid A is known to be a poor substrate of MsbA compared to hexaacylated lipid A, we analyzed the LPS of two Δ(*gmhD–waaA*)-representative derivatives with single-amino-acid substitutions that restore growth at 37 °C in LA rich medium, which is non-permissive for the suppressor-free parental strain. To analyze the lipid A from the suppressor-free Δ(*gmhD–waaA*) strain, such a derivative was grown at 21 °C (permissive growth conditions) in M9 minimal medium, and then shifted to 30 °C for 4 h in LB medium. The mass spectrum of LPS extracted from such a strain revealed a major ion peak at *m/z* 1404.9 Da, corresponding to the tetraacylated lipid IV_A_ precursor (Figure 4a). No additional peaks corresponding to the accumulation of pentaacylated lipid IV_A_, or with any lipid IV_A_ modification, can be observed (Figure 4). It is important to mention here that the suppressor-free Δ(*gmhD–waaA*) strain does not grow at 37 °C, and exhibits bacterial growth arrest followed by cell lysis after 6 h incubation, even at 30 °C, unless extra copies of MsbA are provided, or in the presence of extragenic suppressor mutation. However, when LPS was extracted from SR9919 Δ(*gmhD–waaA*) *msbA* P50S and SR10123 Δ(*gmhD–waaA*) *msbA* R310S grown at 37 °C, their spectra revealed additional mass peaks, aside from the lipid IV_A_ precursor, at *m/z* 1527.9 Da and 1643.1 Da, respectively (Figure 4b,c). Thus, the mass peak at 1527.86 Da corresponds to the addition of *P-EtN*, while the mass peak at 1643.1 Da can be explained as palmitoyl-modified lipid IV_A_ species (Figure 4d). As such modifications occur after translocation of the lipid A, we can conclude that lipid IV_A_ in the presence of chromosomal copies of either MsbA P50S or MsbA R310S variants is efficiently translocated to the OM, compared to that observed in the suppressor-free Δ(*gmhD–waaA*) derivative. Thus, without the overexpression of MsbA, such single-copy MsbA variants can restore the growth of Δ(*gmhD–waaA*) bacteria at 37 °C and support the transport of lipid IV_A_ derivatives. It is again worth noting that these spectra of either the suppressor-free Δ(*gmhD–waaA*) strain or the Δ(*gmhD–waaA*) *msbA* R310S strain also contain ion peaks corresponding to the presence of cardiolipin (C16:0/16:1) (16:0/16:1), with a mass peak at 1348.9 Da.

### 2.5. FtsH Protease Becomes Non-Essential in the ΔwaaA Background at 21 °C

FtsH is an essential protease, and its essentiality is ascribed to its role in regulating LpxC turnover [9,10,11]. However, a Δ*ftsH* derivative can be constructed, either in the presence of hyperactive *sfhC21* alleles of the *fabZ* gene, or when the LPS synthesis is downregulated during its early steps—for example, in *lpxA* or *lpxC* mutants [9,10]. Thus, we wondered if in the absence of WaaA Kdo transferase, Δ*ftsH* could be transduced without any extragenic suppressor mutation. Thus, bacteriophage T4 lysate was prepared on the Δ*ftsH* derivative, which was kept viable due to the *sfhC21* suppressor mutation, and such a lysate was used to transduce into wild-type and Δ*waaA* strains. The results of such transduction clearly establish that while Δ*ftsH* cannot be introduced in the wild-type background, it can be readily introduced into Δ*waaA* when grown at 21 °C (Table 2). As a control, parallel transductions were carried out using the Δ*waaA* strain transformed with the vector, either alone, or in the presence of plasmids expressing the *ftsH* gene (Table 2). These results allow us to conclude that the essential FtsH protease, which regulates LpxC quantity, becomes dispensable in the Δ*waaA* background at low temperatures, which can be ascribed to the reduced total level of LPS in the absence of Kdo transferase.

### 2.6. The Cardiolipin Synthase ClsA Is Essential for the Viability of Strains Lacking Either Three Late Acyltransferases, LpxL, or the Kdo Transferase WaaA and the Conditional Lethality of Δ(lpxM clsA) Bacteria, Which Can Be Suppressed by Mutations in the LPS Transporter MsbA

In the above-described spectra of LPS/glycerophospholipids from either suppressor-free Δ(*gmhD–waaA*) or its derivatives with single-amino-acid suppressor mutations in the *msbA* gene, mass peaks corresponding to the presence of cardiolipin were observed (Figure 2 and Figure 4). This is consistent with our earlier report of the characterization of strains lacking WaaA Kdo transferase [19]. Such observations suggest that strains synthesizing LPS composed of lipid IV_A_ may require cardiolipin in order to sustain their growth. Thus, we performed transduction using either a well-defined *clsA*::Tn*10* or a non-polar Δ*clsA* allele [43] as a donor into isogenic Δ(*gmhD–waaA*) and Δ(*lpxLMP*) strains. Parallel reciprocal transductions by introducing the Δ(*gmhD–waaA*) mutation into the parental wild-type strain, either carrying *clsA*::Tn*10* disruption or with a non-polar Δ*clsA* mutation, were also undertaken. These results revealed that neither *clsA*::Tn*10* nor a Δ*clsA* can be introduced into Δ(*gmhD–waaA*) at 21 °C in M9 minimal medium. Similarly, no viable transductants were obtained from isogenic Δ(*lpxLMP*) derivative at 30 °C in minimal media (permissive growth condition). These genetic studies show that in the absence of Kdo transferase WaaA, the *clsA* gene function is essential for their viability. Furthermore, in strains with intact WaaA Kdo transferase synthesizing LPS with the intact core but with tetraacylated lipid A, as in the case of Δ(*lpxLMP*) strains, the ClsA presence is also essential to sustain their growth in minimal media at 30 °C.

**Figure 5:**
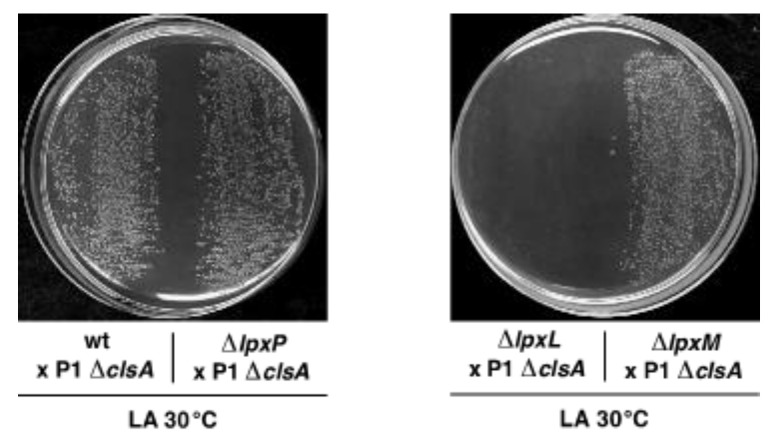
Synthetic lethality of Δ(*lpxL clsA*). Exponentially grown cultures of the wild type and its isogenic deletion derivatives—individually lacking *lpxL*, *lpxM,* and *lpxP*—were used as recipients for bacteriophage P1-mediated transduction in order to introduce the Δ*clsA* mutation. Transductants were plated at 30 °C in LA agar and incubated for 24 h. Data presented are from one of the representative experiments.

Next, we addressed which of the three acyltransferases is required for the growth of a Δ*clsA* strain. As a Δ*lpxL* derivative is temperature sensitive in LA medium above 33 °C, parallel transductions were performed at 30 °C in LA using strains individually lacking genes encoding three acyltransferases, with the wild type as a control (Figure 5). Viable Δ(*lpxM clsA*) and Δ(*lpxP clsA*) transductants were obtained at the same frequency as when Δ*clsA* alone was introduced to the wild type. However, only background growth was visible when Δ(*lpxL clsA*) combination was constructed (Figure 5). However, it should be noted that the colony size of the Δ(*lpxM clsA*) derivative was reduced, although it retained viability at 30 °C—albeit to a reduced extent. Furthermore, Δ(*lpxM clsA*) bacteria obtained at 30 °C were found to be unable to propagate at 37 °C in LA medium, suggesting conditional synthetic lethality. Thus, we can conclude that, at 30 °C in LA medium, LpxL lauroyl acyl chain transferase is required for the viability of a Δ*clsA* strain, and Δ(*lpxL clsA*) combination is synthetically lethal, while as Δ(*lpxM clsA*) bacteria are conditionally lethal at 37 °C.

**Figure 6:**
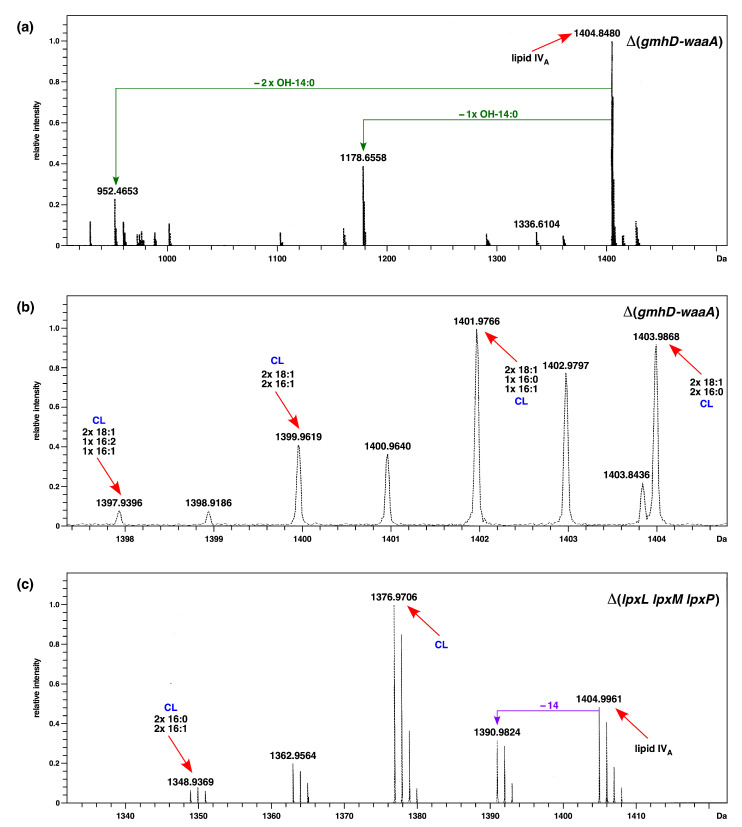
Cardiolipin presence in LPS samples of strains synthesizing LPS with lipid IV_A_ derivatives: (**a**) Fragmentation mass spectra with ion peak at 1404.8 Da corresponding to lipid IV_A_ from the Δ(*gmhD–waaA*) strain grown at 21 °C. (**b**) Fragmentation mass spectra with ion peak at 1403.98 Da, obtained from the Δ(*gmhD–waaA*) strain grown at 21 °C. (**c**) Mass spectra of the lipid A part of LPS obtained from the Δ(*lpxLMP*) strain grown at 30 °C in M9 medium. Charge deconvoluted ESI FT–ICR mass spectra in negative ion mode are presented. The mass numbers refer to monoisotopic peaks with the proposed composition. The mass peaks marked CL correspond to the predicted presence of cardiolipin species with varying acyl chain lengths, with the predicted composition indicated.

To gain further insights into the presence of cardiolipins, we performed high-resolution ESI FT–ICR mass spectrometry, generating ions comprising lipid A and cardiolipin structures with mass peaks predicted to correspond to free lipid IV_A_ (mass peak at 1404.9 Da) and predicted mass peaks corresponding to cardiolipin presence. Under soft ionization conditions, we can confirm that the mass peak at 1404.9 Da represents the tetraacylated 1,4′ bisphosphorylated lipid IV_A_ precursor, while the mass peaks at 1178.6 Da and 952.4 Da can be explained as triacylated and diacylated lipid IV_A_ species, respectively, arising due to ionization (Figure 6a). The fragmentation spectra of ions corresponding to cardiolipin at 1403.9 Da can be explained as cardiolipin with the predicted composition 2 × 18:1–2 × 16:0. Similarly, mass peaks at 1401.9 Da and 1399.9 Da also fit into cardiolipin with the predicted composition of 2 × 18:1–1 × 16:0-1 × 16:1 and 2 × 18:1–2 × 16:1 (Figure 6b). As shown above, ClsA is essential for the growth of a Δ(*lpxLMP*) strain. Thus, we examined the mass spectra of the LPS of such a strain grown in minimal media at 30 °C. Analysis of mass peaks in the range of lipid IV_A_ after fragmentation of native LPS to yield ion peaks corresponding to the lipid A part reveals an ion peak at 1348.9 Da (Figure 6c), which is also present in the spectra of Δ(*gmhD–waaA*) (Figure 2, Figure 3 and Figure 4). This mass peak at 1348.9 Da can be ascribed to cardiolipin species with the predicted composition (C16:0/16:1)(16:0/16:1). Taken together, our mass spectrometric analysis supports the presence of cardiolipin species in the LPS preparations of Δ(*gmhD–waaA*) and Δ(*lpxLMP*) strains, and based on genetic evidence we show that cardiolipin synthase ClsA is required for the growth of strains with tetraacylated lipid A, such as Δ(*gmhD–waaA*), Δ(*lpxLMP*), and Δ*lpxL*.

As Δ(*lpxM clsA*) bacteria were found to be viable at 30 °C, but not at 37 °C and above, we sought extragenic suppressor mutations that could allow their growth up to 43 °C in LA medium. Such chromosomal suppressor mutations were marked and mapped. DNA sequence analysis of such independently obtained suppressors revealed that eleven out of thirteen suppressors carried suppressor mutation in the *msbA* gene (Table 3 and Figure 3). Out of these, the most preponderant mutation leading to a single-amino-acid exchange of Ser120 to Leu (TCA to TTA) was present in three strains (Table 3 and Figure 3). Two strains each showed single-amino-acid exchange of Met160 to Ile (ATG to ATA) and exchange of Asp431 to Tyr (GAT to TAT). Three other strains showed single-amino-acid exchange of Asp498, Ser164 and Val287. Only one suppressor-carrying strain that restored the growth of Δ(*lpxM clsA*) bacteria contained two substitutions—Ile177 to Met and Asn529 to Lys—in the *msbA* gene. Most of these mutations are substitutions in the highly conserved residues, located either in LPS binding/release domains or in the nucleotide binding domain (NBD), and suggest acceleration of the translocation of underacylated lipid A species (Figure 3). This suggestion gains support from our previous isolation of single-amino-acid Asp498 substitution by Val in MsbA, which suppressed the lethality of Δ(*lpxLMP*) and Δ(*waaC lpxLMP*) derivatives [19]. Structural prediction using the Phyre server posits that Asp498Tyr leads to the expansion of cavity volume by 100.008 Å^3^, which could be important for the accelerated translocation of underacylated lipid A species. These results also support a critical role for cardiolipins in assisting LPS transport, as the absence of LpxM alone does not lead to any significant growth defects [19], given the synthetic lethality of Δ(*lpxM clsA*) bacteria at 37 °C and above.

### 2.7. The Absence of EptB in the ΔwaaC Background Confers Ca^++^ Sensitivity, Which Can Be Rescued by the Mutations in Either the basS or the basR Gene That Confer Polymyxin B Resistance

In the absence of WaaC heptosyltransferase, LPS is composed entirely of Kdo_2_-LA_hexa_, which constitutes the minimal LPS structure that can support the growth of *E. coli* under standard laboratory conditions at 37 °C in LA or M9 minimal media without the requirement of any extragenic suppressors [19,47]. However, Δ*waaC* mutants exhibit a temperature-sensitive growth phenotype above 43 °C, the constitutive induction of RpoE-regulated envelope stress response, and severe permeability defects [19]. Due to the constitutive induction of the RpoE stress response, such bacteria incorporate *P-EtN* preferentially on the second Kdo rather than in the lipid A due to hyperinduction of the RpoE-regulated *eptB* gene [19,33,47]. The *eptB* gene encodes the Ca^++^-dependent phosphoethanolamine transferase specific to the second Kdo [30], and a deletion of the *eptB* gene in the Δ*waaC* background is known to confer Ca^++^ sensitivity [30,31]. Taking advantage of the Ca^++^ sensitivity of the Δ(*waaC eptB*) strain, spontaneous Ca^++^-resistant suppressors were isolated at a frequency of 10^−8^ by plating several independent cultures in LA medium supplemented with either 5 mM or 7 mM CaCl_2_. To identify the suppressor mutation(s), independent Ca^++^-resistant isolates were subjected to saturated transposon mutagenesis in order to mark the mutation. In this manner, the Tn*10* tet^R^-marked Ca^++^-resistant suppressing mutation was back-transduced into the SR8649 Δ(*waaC eptB*) strain in order to validate the suppression using bacteriophage T4-mediated transductions. In this manner, 12 independent Ca^++^-resistant isolates were marked by Tn*10*. The position of Tn*10* was obtained via sequencing of the Tn*10* junction on the chromosome, employing inverse PCR-amplified products using the chromosomal DNA from suppressing strains as a template. Out of 12 Ca^++^-resistant isolates, 9 strains were found to contain suppressor mutations linked to the *basS*/*R* operon. Next, the chromosomal DNA of nine suppressors with the Tn*10* insertion linked to the *basS/R* operon served as a template for PCR amplification of the whole *eptA*-*basR/S* locus. DNA sequence analysis revealed that six strains had a single-amino-acid exchange of BasS A20D (GCC to GAC) (Table 1). Three other strains had a single-amino-acid exchange of G53 to V in the *basR* gene (GGG to GTG) (Table 1).

**Figure 7:**
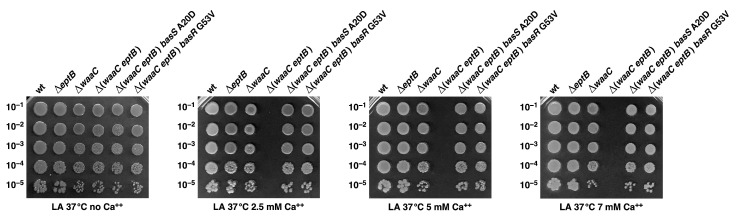
Suppression of the Ca^++^ sensitivity of Δ(*waaC eptB*) by mutations in either the *basS* or the *basR* genes. Exponentially grown cultures of the wild type and its isogenic deletion derivatives of *waaC*, *eptB*, Δ(*waaC eptB*), and strains with the suppressor mutation in either the *basS* or the *basR* genes were adjusted to an OD_595_ of 0.1 and serially spot diluted at 37 °C in LA agar, either supplemented with varying concentrations of CaCl_2_, or without supplementation with CaCl_2_, as indicated. Plates were incubated for 24 h and the data presented are from one of the representative experiments.

Next, we quantified the Ca^++^ resistance of isogenic Δ*eptB*, Δ*waaC*, Δ(*waaC eptB*), Δ(*waaC eptB*) *basS* A20D, and Δ(*waaC eptB*) *basR* G53V strains. The spot dilution assay at various supplementation with CaCl_2_ (2.5 mM, 5 mM, and 7 mM) showed that the removal of EptB alone does not confer sensitivity to Ca^++^, and that Δ*eptB* have a colony-forming ability similar to that of wild-type bacteria even when their growth medium is supplemented with 7 mM CaCl_2_ (Figure 7). However, Δ*waaC* bacteria exhibit a reduction in colony size, although their overall viability is comparable to the wild type (Figure 7). Of major significance are results showing that the suppressor mutations in either the *basS* gene or the *basR* gene restore Ca^++^ resistance to near the wild-type level under the above tested conditions (Figure 7). Without either of these suppressor mutations, Δ(*waaC eptB*) strains exhibit extreme sensitivity, and their growth is abolished by the supplementation of CaCl_2_ at 2.5 mM and above (Figure 7). These results thus reinforce the conclusion that the incorporation of *P-EtN* is essential for the integrity and permeability of the OM in *E. coli*.

### 2.8. Constitutive Induction of BasS/R Regulon due to basS A20D and basR G53V Mutations That Suppress the Ca^++^ Sensitivity of Δ(waaC eptB) Strains

**Figure 8:**
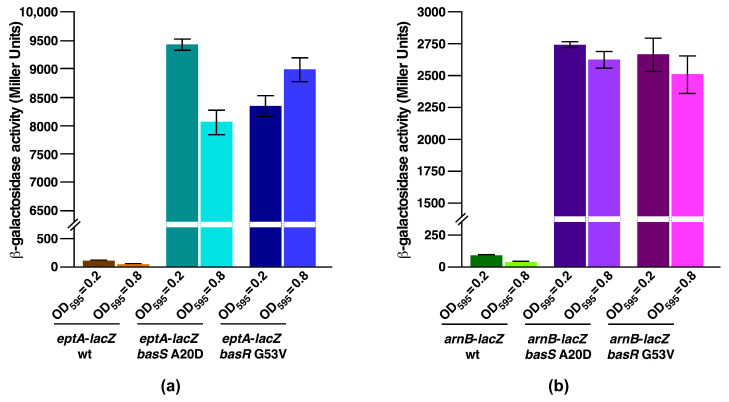
Constitutive induction of *eptA* and *arnB* reporters of the *basS/R* regulon by suppressor mutations that restore the growth of Δ(*waaC eptB*) in media supplemented with CaCl_2_. Exponentially grown cultures of wild type and its derivatives, with mutations in either the *basS* or the *basR* gene carrying either (**a**) *eptA*–*lacZ* or (**b**) *arnB*–*lacZ* single-copy chromosomal promoter fusion, were analyzed for *β*-galactosidase activity. Cultures were adjusted to an OD_595_ of 0.03 and allowed to grow in LB medium at 37 °C. Aliquots of cultures were withdrawn at different growth stages and used to measure the *β*-galactosidase activity. Error bars denote a standard error of three independent measurements.

To address the molecular basis of suppression by BasS and BasR variants, we analyzed the transcriptional activity of two major regulon members of BasS/R. It should be noted that in the lipid A modification system, BasS acts as a sensory histidine kinase, while BasR is the response regulator, and the induction of this two-component system induces transcription of the *eptA* gene and the *arn* regulon [48]. The induction of the BasS/R regulon is known to confer polymyxin B resistance due to the incorporation of non-stoichiometric modifications to the lipid A by *P-EtN*, and by L-Ara4N by EptA and ArnT, respectively. Products of the *eptA* gene and the *arn* operon are involved in the modification of phosphate groups of lipid A by *P-EtN* and L-Ara4N [19,28]. Thus, *basS* A20D or *basR* G53V mutations were introduced into strains carrying *eptA*–*lacZ* and *arnB*–*lacZ* transcriptional fusions and analyzed for *β*-galactosidase activity. Bacterial cultures were grown under conditions that do not incorporate lipid A modifications (LB medium, pH 7.0), without any supplementation with agents that induce the BasS/R regulon. Measurement of *β*-galactosidase activity demonstrated that in the wild-type background *eptA–lacZ* and *arnB–lacZ* fusions are expressed at only basal levels compared to the highly elevated levels in either BasS A20D or BasR G53V derivatives (Figure 8). Moreover, measurement of the *β*-galactosidase activity in BasS A20D background showed that the activity of *eptA*–*lacZ* is induced 82-fold and 162-fold more compared to the isogenic wild type when bacterial cultures were analyzed from an OD_595_ of 0.2 and 0.8, respectively (Figure 8a). Similar results were obtained when the BasR G53V variant was analyzed for activity of the *eptA*–*lacZ* fusion, with an increase of nearly 73-fold and 180-fold at an OD_595_ of 0.2 and 0.8, respectively (Figure 8a). In parallel, measurement of the *β*-galactosidase activity using the chromosomal single-copy *arnB*–*lacZ* fusion showed a 31-fold and 72-fold increase in its activity using aliquots of bacterial cultures with an OD_595_ of 0.2 and 0.8, respectively, with a BasS A20D variant (Figure 8b). Under the same growth conditions, a similar activation of the *arnB*–*lacZ* promoter fusion was observed in the BasR G53V background (Figure 8b). Taken together, these results establish that an extragenic suppressor mutation in either the *basS* gene or the *basR* gene that abolishes the Ca^++^ sensitivity of Δ(*waaC eptB*) derivatives causes the constitutive activation of BasS/R regulon members, as shown by the activity of *eptA* and *arnB* promoters.

### 2.9. A Single-Amino-Acid Suppressor basS A20D Mutation Induces Modification of Lipid A by P-EtN and L-Ara4N

**Figure 9:**
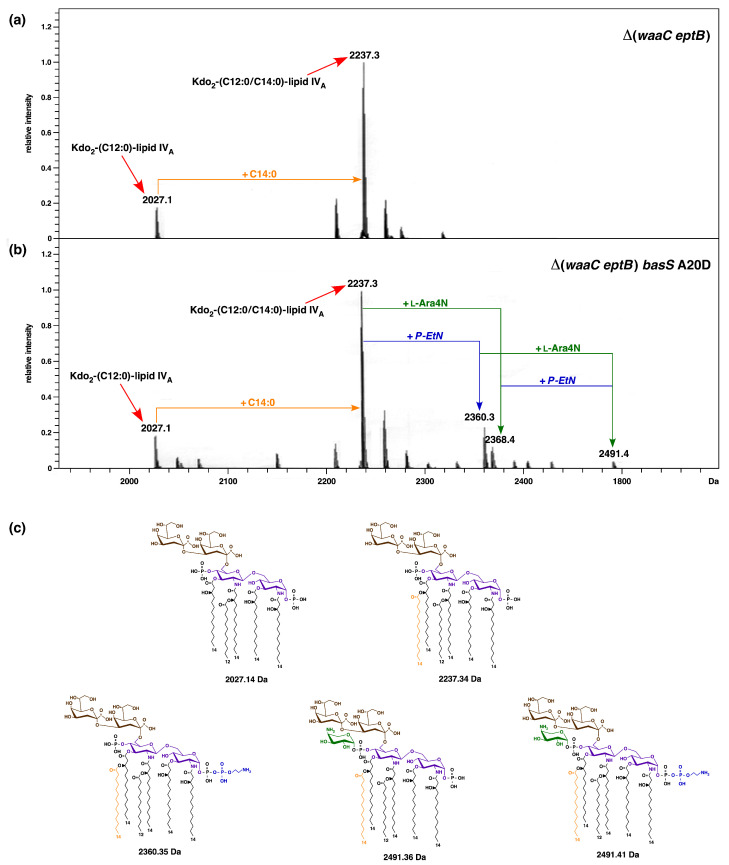
*basS* A20D suppressor mutation of the Ca^++^ sensitivity of Δ(*waaC eptB*) induces the incorporation of lipid A modification by *P-EtN* and L-Ara4N, thereby compensating for the absence of *P-EtN* on the second Kdo due to the lack of EptB phosphoethanolamine transferase, even under conditions where lipid A modifications are not incorporated. (**a**) Mass spectra of LPS obtained from the Δ(*waaC* *eptB*) strain grown at 37 °C in LB medium. (**b**) Mass spectra of LPS obtained from the isogenic Δ(*waaC eptB*) *basS* A20D strain grown at 37 °C in LB medium. Charge-deconvoluted ESI FT–ICR mass spectra in negative ion mode are presented. The mass numbers refer to monoisotopic peaks with the proposed composition. Incorporation of *P-EtN* and L-Ara4N in the lipid A is indicated when *basS* A20D suppressor mutation is present. (**c**) Schematic drawing of the predicted chemical structures of various lipid A derivatives, with or without the modifications by *P-EtN* and L-Ara4N identified in panels **a** and **b,** with predicted mass numbers, are shown.

The lipid A part of LPS from *E. coli* K-12 strains usually does not show the incorporation of non-stoichiometric modification unless the bacteria are exposed to conditions that induce the BasS/R regulon, such as a shift to acidic pH, high Fe^+++^ concentration, or when grown in phosphate-limiting media supplemented with zinc and iron [19,28]. The BasS/R regulon activation is known to induce transcription of the *eptA* and *arn* genes, whose products are involved in the modification of lipid A phosphate residues by *P-EtN* and L-Ara4N, respectively. As shown in the above sections, BasS A20D suppression of Δ(*waaC eptB*) Ca^++^ sensitivity confers a constitutive induction of BasS/R regulon members, such as the *eptA* and *arnB* genes. Thus, we analyzed the LPS of isogenic Δ(*waaC eptB*) and Δ(*waaC eptB*) *basS* A20D strains via mass spectrometry. For these experiments, bacterial cultures were grown in LB medium and used for LPS extraction. Examination of charge-deconvoluted mass spectra in the negative ion mode of LPS of both strains revealed a common mass peak at 2237.3 Da, which corresponds to a typical hexaacylated lipid A with two Kdo residues (Figure 9a). The additional common mass peak at 2027.1 Da can be explained as the accumulation of pentaacylated Kdo_2_-lipid A derivatives lacking the myristoyl chain. However, of specific interest are the presence of mass peaks at 2360.3 Da and 2368.4 Da in the spectra of strain Δ(*waaC eptB*) *basS* A20D, which are absent in Δ(*waaC eptB*) without the suppressor mutation (Figure 9b). These mass peaks correspond to the addition of *P-EtN* and L-Ara4N, respectively, to Δ(*waaC eptB*) *basS* A20D. The mass peak at 2491.4 Da is also present, reflecting the incorporation of both *P-EtN* and L-Ara4N in Δ(*waaC eptB*) *basS* A20D in the lipid A, but is again absent in the parental Δ(*waaC eptB*) strain without the *basS* suppressor mutation. Thus, these results allow us to conclude that the constitutive induction of the BasS/R regulon that induces the incorporation of *P-EtN* and L-Ara4N can compensate for the absence of *P-EtN* on the second Kdo due to the absence of EptB phosphoethanolamine transferase. This compensatory addition of *P-EtN* to the lipid A also explains the suppression of the calcium sensitivity of Δ(*waaC eptB*) strains, and provides the molecular basis of Ca^++^ sensitivity.

### 2.10. Overexpression of the eptA Gene Can Alleviate the Ca^++^ Sensitivity of Δ(waaC eptB) Bacteria

**Figure 10:**
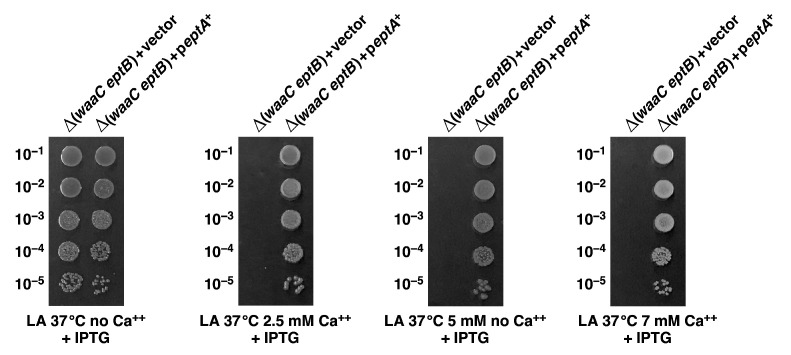
Overexpression of the *eptA* gene restores the growth of Δ(*waaC eptB*) strains in Ca^++^-supplemented media. Exponentially grown cultures of Δ(*waaC eptB*) carrying either the vector alone or the plasmid with the inducible expression of the *eptA* gene were adjusted to an OD_595_ of 0.1 and serially spot diluted at 37 °C in LA agar, either supplemented with varying concentrations of CaCl_2_, or without supplementation with CaCl_2_, as indicated. Plates were incubated for 24 h, and the data presented are from one of the representative experiments.

In the above sections, we showed that BasS A20D and BasR G53V alterations in a single copy can restore the wild-type-like growth of Δ(*waaC eptB*) bacteria in Ca^++^-supplemented growth media. This suppression was explained on the basis of constitutive induction of the expression of the *eptA* and *arn* genes, which in turn leads to the modification of lipid A by *P-EtN* and L-Ara4N even under growth conditions that do not lead to such substitution in the lipid A part. We rationalized the suppression of Ca^++^ sensitivity by compensation for the lack of *P-EtN* on the second Kdo by its incorporation in the lipid A. Thus, we elaborated on this suppression by performing experiments with the ectopic expression of the *eptA* gene from an inducible promoter on a plasmid. Thus, a Δ(*waaC eptB*) bacterial strain was transformed, either with the vector alone or with the plasmid expressing the *eptA* gene, and such isogenic strains were tested for their ability to grow in Ca^++^-supplemented growth media via the spot dilution assay. The expression of the *eptA* gene was induced by the addition of 75 μM IPTG, when the growth media were supplemented with varying concentrations of CaCl_2_. The data presented in Figure 10 show that in the presence of the vector alone Δ(*waaC eptB*) bacteria exhibit sensitivity to CaCl_2_. However, when the plasmid expressing the e*ptA* gene was used, normal growth was restored to Δ(*waaC eptB*) bacteria. Thus, these data convincingly show that in the absence of the addition of EptB-dependent *P-EtN* to the second Kdo, modification of lipid A by the EptA-dependent *P-EtN* incorporation is sufficient to suppress the Ca^++^ sensitivity of Δ(*waaC eptB*) bacteria. Furthermore, the main reason for the Ca^++^ sensitivity of Δ(*waaC eptB*) bacteria is the lack of *P-EtN* substitution.

### 2.11. Constitutive basS/R Suppressor Mutations Confer Polymyxin B Resistance

**Figure 11:**
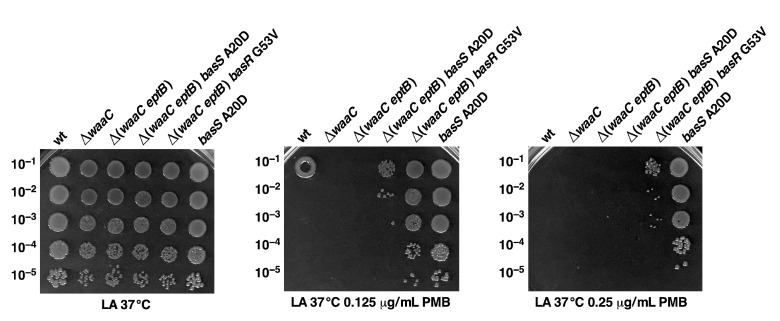
Suppressor mutations in either the *basS* or *basR* genes confer polymyxin B resistance. Exponentially grown cultures of the wild type, its isogenic derivatives in the absence of the *waaC* gene, Δ(*waaC eptB*)*,* and its derivatives with the suppressor mutation in either the *basS* or the *basR* genes, were adjusted to an OD_595_ of 0.1 and serially spot diluted at 37 °C, either in in LA agar alone, or supplemented with either 0.125 μg/mL or 0.25 μg/mL of polymyxin B. Additionally, the culture of wild-type strain carrying *basS* A20D is included (last lane). Plates were incubated for 24 h, and the data presented are from one of the representative experiments. Note polymyxin B resistance when the suppressor mutation mapping to either the *basS* or *basR* genes is present.

In the above experiments, we showed that BasS A20D and BasR G53V can suppress the Ca^++^ sensitivity of Δ(*waaC eptB*) mutant bacteria, and based on the measurement of the activity of the *arnB*–*lacZ* and *eptA*–*lacZ* reporter systems, such mutations lead to the constitutive activation of the BasS/R regulon. Furthermore, we also showed that these suppressor mutations lead to the incorporation of *P-EtN* and L-Ara4N in the lipid A, even under growth conditions where wild-type bacteria do not show any such lipid A modifications. These modifications are known to confer resistance to cationic antimicrobial peptides, such as polymyxin B [28]. Thus, isogenic derivatives of Δ(*waaC eptB*), with or without suppressor mutations, were tested for sensitivity in growth media supplemented with different concentrations of polymyxin B, using the spot dilution assay. As controls, the parental wild-type strain and its Δ*waaC* derivative were also included. These spot dilution experiments revealed that the wild type, Δ*waaC*, and Δ(*waaC eptB*) exhibit sensitivity to polymyxin B, but Δ(*waaC eptB*) *basS* A20D and Δ(*waaC eptB*) *basR* G53V are partially resistant to polymyxin B (Figure 11). We further investigated whether a suppressor mutation that confers a constitutive induction of the BasS/R regulon could result in elevated resistance to polymyxin B in wild-type bacteria. Thus, *basS* A20D was transduced into the parental wild-type strain and tested alongside other strains. This analysis revealed that the introduction of *basS* A20D mutation confers polymyxin B resistance in the otherwise wild-type strain (Figure 11). Polymyxin B resistance in the wild-type background with *basR* G53V mutation was not evaluated in this experiment, since its resistance has already been documented [49]. Thus, in summary, we can conclude that a *basS*/*R* constitutive mutation confers polymyxin B resistance in the wild type, and also elevates resistance to such antibiotics in Δ(*waaC eptB*) bacteria as well as suppressing Ca^++^ sensitivity.

### 2.12. The Absence of WaaF, but Not That of WaaG or WaaP, also Confers Sensitivity to Ca^++^ When EptB Is Simultaneously Absent

**Figure 12:**
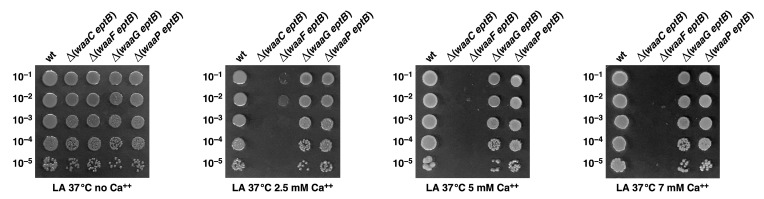
Δ(*waaC eptB*) and Δ(*waaF eptB*) bacteria exhibit sensitivity to Ca^++^, but Δ(*waaG eptB*) and Δ(*waaP eptB*) combinations behave like the wild type. Exponentially grown cultures of the wild type, its isogenic derivatives lacking the *eptB* gene, and one of the early LPS inner core biosynthetic enzymes were adjusted to an OD_595_ of 0.1 and serially spot diluted at 37 °C, either in LA agar alone, or supplemented with different concentrations of CaCl_2_. Plates were incubated for 24 h, and the data presented are from one of the representative experiments.

We also tested whether the conditional lethality observed in Δ(*waaC eptB*) strains, when challenged with a sublethal concentration of Ca^++^, was unique, or if the lack of EptB with deletion derivatives of genes encoding other inner core LPS biosynthetic enzymes could also confer Ca^++^ sensitivity. Thus, panels of isogenic deletion derivatives Δ(*waaF eptB*), Δ(*waaG eptB*)*,* and Δ(*waaP eptB*) were constructed via bacteriophage P1-mediated transductions. The sensitivity of such isogenic deletion derivatives combined with *eptB* deletions was compared in LA medium supplemented with various concentrations of CaCl_2_. The data presented reveal that in addition to Δ(*waaC eptB*), Δ(*waaF eptB*) also exhibits extreme sensitivity to supplementation of the growth medium with CaCl_2_ (Figure 12) However, the viability of Δ(*waaG eptB*) and Δ(*waaP eptB*) strains was comparable to that of the parental wild-type strain (Figure 12). These results are consistent with the previously observed near-exclusive incorporation of *P-EtN* on the second Kdo in the Δ*waaC* and Δ*waaF* derivatives [29], and hence, the importance of the incorporation of *P-EtN* on the Kdo in such strains in combating CaCl_2_ sensitivity.

### 2.13. Relief of Translational Repression of EptB in the Absence of MgrR sRNA Abrogates the P-EtN Incorporation in the Lipid A

**Figure 13:**
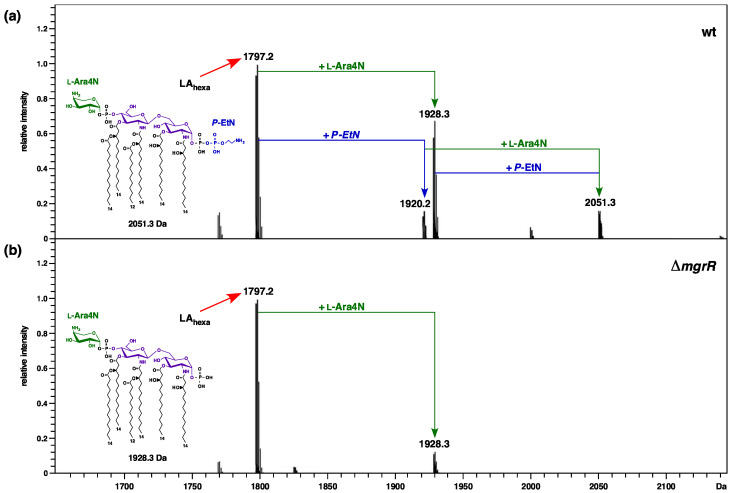
Δ*mgrR* strains do not incorporate lipid A nonstoichiometric modification by *P-EtN* on lipid A, even under growth conditions that induce lipid A and LPS core modifications. Charge-deconvoluted ESI FT—ICR mass spectra in negative ion mode are presented. (**a**) Mass spectra of the lipid A part of the LPS obtained from the wild-type strain grown at 37 °C in phosphate-limiting 121 medium—conditions that favor the incorporation of lipid A modifications. (**b**) Mass spectra of LPS obtained from the isogenic Δ*mgrR* strain, also grown at 37 °C in phosphate-limiting 121 medium. The mass numbers refer to monoisotopic peaks with the proposed composition. Only mass peaks corresponding to the lipid A part are marked, and the substitutions with *P-EtN* and L-Ara4N are indicated.

We previously reported the mass spectrometric analysis of core oligosaccharides obtained from the native LPS of a strain lacking the MgrR sRNA, compared to the isogenic deletion derivatives of various envelope stress regulatory genes [33]. The MgrR sRNA is known to repress translation of the *eptB* mRNA [32], which can prevent the incorporation of *P-EtN* on the second Kdo. However, the impact of the deletion of MgrR sRNA on the incorporation of non-stoichiometric modifications by *P-EtN* and L-Ara4N in the lipid A part has not been reported. Given the above-described importance of *P-EtN* incorporation on the second Kdo to the cell envelope stress response, we analyzed native LPS obtained from growth conditions that induce lipid A and LPS core modifications from Δ*mgrR* and its parental wild-type strain. Purified LPS was analyzed using high resolution ESI FT–ICR MS after unspecific fragmentation, generating ions comprising heterogenous lipid A part structures, as previously described [19]. As expected, the lipid A part of the LPS obtained from the wild-type strain and from its Δ*mgrR* derivative revealed a common characteristic mass peak at 1797.2 Da, corresponding to hexaacylated 1,4′ bisphosphorylated species. In the lipid spectra of the wild-type strain, additional molecular ions that are conspicuous can be ascribed to the incorporation of *P-EtN* (1920.2 Da), L-Ara4N (1928.3 Da), and both substitutions (2051.3 Da) (Figure 13). Of interest, however, there are specific differences in the incorporation of non-stoichiometric modifications in the lipid A part of the Δ*mgrR* derivative. These differences are manifested in the lack of mass peaks that correspond to the incorporation of *P-EtN* in the Δ*mgrR* derivative (absence of mass peaks at 1920.2 Da and 2051.3 Da). However, it should be noted that the mass peak corresponding to the incorporation of L-Ara4N (1928.3 Da) is present in the spectra of the lipid A part of Δ*mgrR*, quite like that observed in the lipid A part of the wild-type strain (Figure 13). Thus, these results allow us to conclude that genetic backgrounds that cause either transcriptional upregulation of the *eptB* gene (Δ*waaC*) or silencing of the translational repression of *eptB* mRNA (lack of MgrR sRNA)—which also leads to the increased expression of *eptB*—result in the preferential incorporation of *P-EtN* on the second Kdo at the expense of *P-EtN* modification of the lipid A part. Thus, these results establish that, in the absence of MgrR sRNA, the preferential incorporation of *P-EtN* occurs on the second Kdo, and under such conditions lipid A phosphate groups are not modified by *P-EtN*.

### 2.14. A Single-Amino-Acid Suppressor Mutation in OppA Alleviates Permeability Defects of ΔwaaC and Allows a Deletion of the surA Gene in the Absence of WaaC Heptosyltransferase

**Figure 14:**
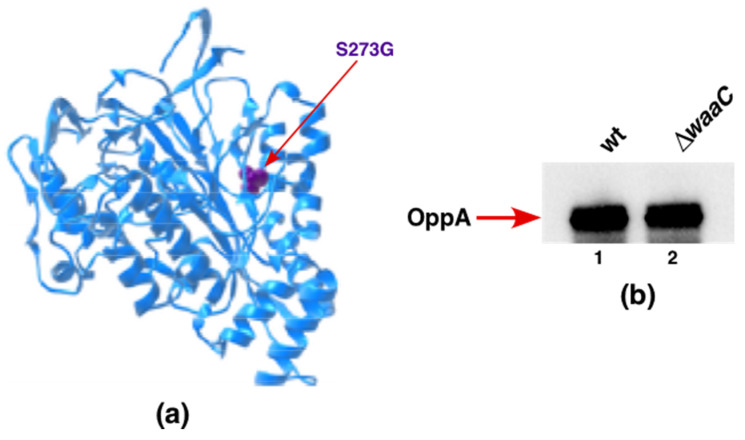
A single-amino-acid alteration to OppA S273 overcomes the synthetic lethality of Δ(*waaC surA*) bacteria. (**a**) Position of S273 in the structure of OppA (PDB 3TCH). (**b**) Immunoblot analysis of total cellular extracts obtained from the wild type and its Δ*waaC* derivative, carrying a single-copy 3xFLAG appended to the C-terminal end of OppA, were grown in LB medium at 37 °C. Equivalent amounts of proteins were applied to a 12.5% SDS–PAGE, and resolved proteins were transferred by Western blotting. Immunoblots were treated with an M2 monoclonal antibody to reveal OppA–3xFLAG. The arrow indicates the position of OppA.

Although the *waaC* gene encoding heptosyltransferase I is not essential under standard laboratory growth conditions, it becomes essential if its deletion derivatives are grown at temperatures above 43 °C, quite like when *gmhD* (*htrM*) is absent, which synthesizes LPS composed solely of Kdo_2_-LA_hexa_ [19,50]. Furthermore, Δ*waaC* derivatives exhibit synthetic lethality when the major periplasmic folding catalyst SurA is also absent [19]. Thus, suppressor analysis was executed to identify whether a mutation in any gene(s) could circumvent the synthetic lethality of Δ(*waaC surA*) combination. To isolate suppressors, multiple rounds of transductions were carried out in order to obtain Δ(*waaC surA*)-viable transductants. Such transductants were marked with Tn*10* in order to map and identify a suppressor mutation(s), which allows the growth of Δ(*waaC surA*) as described in the Materials and Methods section. One such Tn*10*-linked suppressor mutation, linked more than 90% to the *clsA* gene (encoding cardiolipin synthase [43]), was found to breed true. To directly identify the suppressor mutation, the chromosomal DNA of the Δ(*waaC surA*) strain was sequenced using appropriate oligonucleotides covering the region around the location of the Tn*10* from the suppressing strain. Examination of the DNA sequencing revealed a single-amino-acid alteration in OppA, resulting in the replacement of Ser273 (AGC) by Gly (GGC). These results were further validated by transductions using either the Δ*waaC oppA* S273G strain or Δ*waaC* alone as a recipient for introducing a deletion of the *surA* gene (Table 4). Viable Δ(*waaC surA*) transductants could be only obtained when the *oppA* S273G suppressor mutation was present (Table 4). Modeling of Ser273 amino acid substitution by Gly residue in the structure of OppA (Figure 14a), using PDB 3TCH as a template, suggests that this substitution can lead to the expansion of OppA’s cavity volume by 74.52 Å using the Phyre server [51].

Next, we examined the levels of OppA in Δ*waaC* compared to the parental wild type, using isogenic strains with a chromosomal 3xFLAG appended in-frame at the C-terminal end. Immunoblot analysis using an anti-FLAG monoclonal antibody revealed nearly unchanged amounts of OppA–FLAG when the *waaC* gene was deleted, compared to the wild type with the intact WaaC (Figure 14b).

OppA is a periplasmic oligopeptide-binding protein, and a potential chaperone-like function has been ascribed to it [52,53]. Thus, we tested whether overexpression of OppA could also bypass the synthetic lethality of Δ(*waaC surA*). Such transduction experiments revealed that Δ(*waaC surA*) cannot be constructed if *oppA* expression is induced. Alternatively, if Gly273 is a loss-of-function mutation, then a Δ(*waaC surA*) derivative should be viable when the *oppA* gene is deleted. Contrary to this hypothesis, the Δ(*waaC surA*) combination is lethal whether the *oppA* gene is deleted or its expression is induced. Taken together, our results suggest that the OppA S273G substitution is a gain-of-function mutation that can explain its isolation as an extragenic suppressor preventing the synthetic lethality of Δ(*waaC surA*) combination.

### 2.15. Overexpression of GcvB sRNA Represses LpxC Amounts in the Wild Type and Suppresses ΔlapAB Lethality

LapB is essential for bacterial viability due to its critical role in the regulated proteolysis of LpxC [9,12]. LpxC quantities are regulated by the LapB/FtsH/LapC complex, and at elevated temperatures by HslUV protease [9,12,13]. However, in *E. coli*, overexpression of SlrA sRNA, or the absence of Lpp lipoprotein or genetic backgrounds that reduce the total LPS content, can allow the growth of strains with deletion of *lapAB* genes [9]. Up to now, the role of regulatory small RNAs other than SlrA (MicL) in the regulation of LpxC quantities has not been addressed [9]. Hence, we systematically overexpressed the best characterized sRNAs other than SlrA (MicL), and examined whether *lapAB* genes could be deleted when a candidate sRNA is overexpressed. Among sRNAs, we chose to further investigate the role of GcvB sRNA, as it regulates the expression of the largest number of mRNAs in *E. coli* and *Salmonella* [35,36]. Thus, the *gcvB* gene was cloned in the previously described pBR322-based plasmid [43], where expression was controlled from the inducible *lac* promoter. Thus, a bacteriophage P1 lysate grown on the Δ*lapAB* strain in M9 minimal medium at 30 °C (permissive growth conditions) was used to transduce this mutation into isogenic strains harboring either the pBR322 vector alone or the plasmid carrying *gcvB*, in the presence of 75 μM IPTG, in order to induce the expression of *gcvB* sRNA. The plasmid expressing the SlrA sRNA served as a positive control. Transductants were plated at 30 °C and 37 °C in M9 and LA media. The results presented in Table 5 show that a Δ*lapAB* mutation can be introduced at 37 °C, when either SlrA or GcvB sRNAs are overexpressed, but not when the vector alone is present. As another control, in the same set of transductional experiments, we were able to introduce a *lppA*::Tn*10* disruption in the Δ*lapAB* strain. These results are consistent with the earlier discovery of SlrA acting as a repressor of *lpp* mRNA translation [9] and the identification of *lppA*::Tn*10* as preventing lethality in the absence of LapAB proteins [9]. However, the known in vivo mRNA targets of GcvB and SlrA (MicL) are different [37,54,55], and thus suppression is likely to operate via a different mechanism.

**Figure 15:**
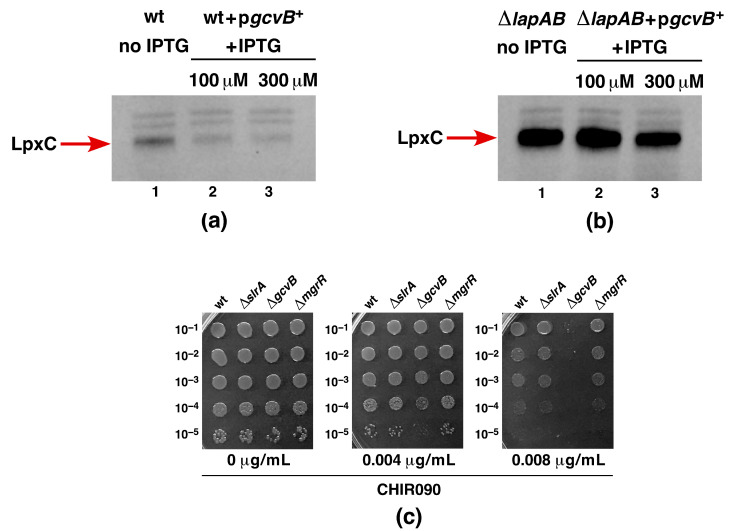
Overexpression of the *gcvB* sRNA represses LpxC accumulation in the wild type. (**a**) Equivalent amounts of total cellular proteins were applied from the isogenic strain’s wild type with the vector alone (lane 1) and when the expression of *gcvB* was induced upon the addition of 100 and 300 μM IPTG (lanes 2 and 3, respectively). Proteins were resolved on a 12.5% SDS–PAGE and immunoblotted with anti-LpxC polyclonal antibodies. (**b**) Immunoblot analysis of the total cellular extracts obtained from Δ*lapAB* with the vector alone without the addition of IPTG (lane 1) and from Δ*lapAB* transformed with the plasmid with the inducible expression of *gcvB* sRNA (lanes 2 and 3) with the addition of the indicated amounts of IPTG. Cultures of Δ*lapAB* were grown in M9 minimal medium up to an OD_595_ of 0.2 prior to the addition of IPTG, and allowed to grow for another 2 h. Equivalent amounts of proteins were resolved on a 12.5% SDS–PAGE and transferred using Western blotting. The immunoblots were treated with anti-LpxC polyclonal antibodies in order to detect LpxC. The arrow indicates the position of LpxC. (**c**) Exponentially grown cultures of the wild type and its deletion derivatives lacking sRNA encoding genes were serially spot diluted in LA agar, supplemented with or without varying concentrations of CHIR090, as indicated.

Next, we analyzed the impact of GcvB overexpression on the quantities of LpxC in the wild type and in its isogenic Δ*lapAB* derivative by immunoblotting. These experiments revealed that LpxC quantities are reduced when GcvB expression is induced in the wild type at different levels of concentrations of IPTG (100 μM and 300 μM, respectively) (Figure 15). However, when the equivalent amount of total cell lysate from Δ*lapAB* was analyzed using Western blotting, the LpxC amount was only marginally reduced at the higher concentration of the inducer of GcvB expression (Figure 15). It should be noted that Δ*lapAB* strains accumulate LpxC in excess due to defects in proteolysis, and translational repression of LpxC by GcvB may not be easily discernable [9]. If GcvB is indeed involved in regulating LpxC synthesis, one should expect strains lacking this sRNA to have altered sensitivity to CHIR090, which is a known inhibitor of LpxC [12]. Thus, we investigated the sensitivity profiles of the wild-type strain and its isogenic Δ*slrA*, Δ*gcvB*, and Δ*mgrR* derivatives to varying concentrations of CHIR090, as described earlier [12]. As shown in Figure 15c, among these strains, only a Δ*gcvB* strain is sensitive to CHIR090, with growth abolished at 0.008 μg/mL supplementation of CHIR090 to the growth medium—a concentration that is tolerated by other isogenic strains. This is thus far the first deletion strain of any non-coding sRNA gene that confers sensitivity to CHIR090, reinforcing our results indicating a role for GcvB in regulating LpxC expression. Taken together, we can conclude that overexpression of GcvB sRNA significantly reduces the LpxC quantities in the wild type, with a mild effect on Δ*lapAB*, and can explain the reduction in lethality in the absence of LapAB proteins.

## 3. Discussion

It is well established that, in *E. coli* K-12, LPS is essential for bacterial viability, and the minimal structure of LPS that can support bacterial viability under standard laboratory growth conditions is Kdo_2_-LA_hexa_. However, suppressor-free strains that synthesize only Kdo_2_-lipid IV_A_, or with only the lipid IV_A_ precursor, can be constructed, but with a growth restricted to slow-growth conditions of minimal media around 21–23 °C [19]. The limited growth of strains synthesizing LPS of lipid IV_A_ derivatives is consistent with the relatively poor translocation of tetraacylated LPS compared to hexaacylated LPS, as well as the essentiality of WaaA Kdo transferase under normal growth conditions [18,19,25]. Thus, unsurprisingly, overproduction of MsbA LPS flippase can overcome the growth defects of strains synthesizing lipid IV_A_ LPS [19,25]. *E. coli* K-12 also expresses another Kdo transferase—WaaZ—which is non-essential for bacterial viability, and transfers a third Kdo, which is attached to the lipid A-anchored Kdo disaccharide [33]. Interestingly, the Δ*waaZ* bacteria exhibit conditional synthetic lethality in strains synthesizing lipid IV_A_ LPS when the *lpxL, lpxM*, and *lpxP* genes are simultaneously absent [33]. Thus, it becomes imperative to construct strains that lack both the Kdo transferase WaaA and the entire *waa* locus, which also contains the *waaZ* gene. The *waa* locus is comprised of three operons that include genes encoding all glycosyltransferases, *O*-antigen ligase, heptose kinases, and also two Kdo transferases [56]. Thus, in this study, we explored the possibility of constructing suppressor-free derivatives with deletions spanning from *waaF* to *waaA* and *gmhD*–*waaA*, thereby removing the entire *waa* locus. Indeed, we were able to construct such strains, and verified them via transduction into different wild-type strains—such as W3110, MG1655, and BW25113—with viable transductants at 21 °C. No transductants were obtained at 30 or 37 °C, either in M9 minimal medium or in LA medium. Furthermore, transduction frequency and colony size were severely diminished even in LA medium at 21 °C, unlike the higher number of viable transductants in the M9 minimal medium. Thus, the strains constructed are the first of their kind, and the lack of Kdo was ascertained by the absence of cross-reactivity to the Kdo-specific monoclonal antibody A20 [57], and by analysis of the LPS composition using mass spectrometry. This allowed us to conclude that the entire *waa* locus is dispensable under the slow growth of minimal media at 21 °C, without any requirement for extragenic suppressor mutations or extra copies of MsbA. Of interest are the data from mass spectrometry that showed that Δ(*gmhD–waaA*) bacteria synthesize the glycosylation-free lipid IV_A_ precursor—which can serve in vivo as an acceptor for late acyltransferases—when such bacteria are grown in slow-growth conditions at low temperatures in either M9 medium or LB medium. Of significance are the presence of mass peaks that correlate with the incorporation of acyl carrier protein-dependent palmitoleate (C16:1) and lauroyl (C12:0) acyl chains in the lipid IV_A_ precursor. Further conversion of lipid IV_A_+palmitoleate into hexaacylated lipid by the addition of the myristoyl acyl chain is also observed under slow-growth conditions of 21 °C. These data convincingly allow us to draw the conclusion that late acylation steps mediated by LpxL, LpxM, and LpxP can occur even in the absence of Kdo at 21 °C, with the lipid IV_A_ precursor serving as an acceptor. These results were further corroborated by the absence of secondary acyl chain incorporation when the *lpxL*, *lpxM*, and *lpxP* genes were deleted in the Δ(*gmhD*–*waaA*) background. Furthermore, Δ(*gmhD*–*waaA lpxL lpxM lpxP*) derivatives could be constructed at the same efficiency in M9 minimal medium at 21 °C as a Δ*waaA* derivative. Thus, neither acyl–oxyl chains within the lipid A nor the Kdo attachment to the lipid A are essential for bacterial viability under slow-growth conditions in M9 medium at 21 °C. These results are consistent with our earlier construction of suppressor-free Δ*waaA* derivatives synthesizing only lipid IV_A_ derivatives [19]. However, unlike Δ(*gmhD*–*waaA*) bacteria, a Δ(*gmhD*–*waaA lpxL lpxM lpxP*) derivative is viable only in M9 medium but not in LA medium at 21 °C without any suppressors. Although other studies have also reported *E. coli* strains containing LPS composed of lipid IV_A_ derivatives, their growth required extragenic suppressors, and they were constructed either not in *E. coli* K-12 but *E. coli* B—which has additional mutations in BasS/R and PhoB/R and is of a different core type—or in *E. coli* K-12 with constitutive induction of BasS/R and with extra copies of MsbA/LpxL [25,26]. Thus, the Δ(*gmhD*–*waaA*) and Δ(*gmhD*–*waaA lpxL lpxM lpxP*) strains described here, without any suppressors, are the first of their kind.

For the survival of the bacteria, lipid IV_A_ must be translocated to the outer membrane, particularly under fast-growing conditions. However, tetraacylated lipid IV_A_ is not an efficient substrate for the MsbA lipid A transporter [18,19,25]. Thus, we sought suppressors of Δ(*gmhD*–*waaA*) strains that could support growth between 32 and 37 °C in LA medium. Indeed, two independent single-copy exchanges in MsbA, leading to the substitution of either Pro50 to Ser or R310 to Ser, were identified, which allowed either Δ*waaA* or Δ(*gmhD*–*waaA*) to grow at up to 37 °C in LA medium. Modeling of these MsbA alterations to recently described structures of MsbA [44,45,46] leads us to make significant observations. The amino acid residue R310 is located in the TM6 and in the inward-facing MsbA structure; two portals on the opposite side are formed by TM4 and TM6, which are predicted to facilitate the entry of lipid A by lateral diffusion [46]. More importantly, R310 in TM6 is a part of the MsbA structural element lined with positively charged residues, and could interact with negatively charged residues in the lipid A. Substitution of Arg310 by Ser could alter the selectivity of MsbA for tetraacylated lipid IV_A_. Additional support comes from the MsbA structure resolved in complex with G902 and G907 inhibitors, which revealed that the inner vestibule of MsbA, where R310 is part of TM6, provides structural features that define MsbA’s hydrocarbon ruler selection for 12-carbon and 14-carbon acyl chains of lipid A, where basic residues could form a selective filter around P-GlcN residues of lipid A [44]. Further studies will be needed to measure the in vitro binding ability of MsbA R310S compared to wild-type MsbA, using tetraacylated and hexaacylated lipid A species.

We can similarly speculate that MsbA P50S substitution could confer relaxed specificity for more efficient translocation of tetraacylated lipid A. In the crystal structure, MsbA P50 residue is located in the putative lipid A-binding site on the periplasmic surface of MsbA [46]. This lipid A-binding site, wherein P50 residue is located, is positioned above a shallow surface grove formed at the periplasmic ends of TM1, TM2, and TM3, which could provide an exit portal for the lipid A. Contact of P50 amino acid with acyl chains of lipid A can be observed in these structures [46]. Analysis of the composition of LPS of Δ(*gmhD*–*waaA*) *msbA* R310S and Δ(*gmhD*–*waaA*) *msbA* P50S revealed that their lipid IV_A_ precursor shows non-stoichiometric modification by *P-EtN* and the incorporation of the C16:0 plamitoly chain. The secondary palmitate chain is added at position 2′ on the proximal glucosoamine [58]. As this modification requires the OM-located PagP enzyme, which uses glycerophospholipids as an acyl donor [58], this clearly indicates that in the presence of such an MsbA suppressor mutation the lipid IV_A_ precursor has been translocated to the OM. Another finding that supports a more efficient translocation of lipid IV_A_ species in the presence of suppressors mapping to the *msbA* gene is the modification by *P-EtN*. This modification also occurs after the ranslocation of lipid A, as the active site of the EptA enzyme is located on the periplasmic side [28]. These lipid A modifications often serve as markers for lipid A translocation [19,25,28]. Thus, our results provide the rationale for the explanation of the isolation of the Δ(*gmhD*–*waaA*) *msbA* R310S and Δ(*gmhD*–*waaA*) *msbA* P50S suppressor strains.

Based on the observed increased accumulation of cardiolipin species in both Δ(*gmhD*–*waaA*) and Δ(*lpxLMP*), we further investigated their significance. *E. coli* contains three cardiolipin synthases—namely, ClsA, ClsB, and ClsC [35]. Among these, ClsA contributes the majority of the cardiolipin, and is responsible for nearly all of its synthesis in the log phase [35,59]. Thus, we further investigated the role of ClsA in strains synthesizing tetraacylated lipid A. Importantly, cardiolipin species that we identified in this work in the mass spectra of lipid A/glycerophospholipids obtained from Δ(*gmhD*–*waaA*) or Δ(*lpxLMP*) strains were identical to those reported earlier in either *E. coli* or *Salmonella* [35,60]. Based on genetic analysis, we can show that ClsA is essential for the growth of Δ(*gmhD*–*waaA*) bacteria at 21 °C, as well as for the growth of either the Δ*lpxLMP* or Δ*lpxL* strains under growth conditions that are usually permissive for such bacteria. Δ*lpxLMP* and Δ*lpxL* strains primarily synthesize LPS composed of tetraacylated lipid A with an intact core [19]. Similarly, ClsA was also found to be needed for the growth of Δ*lpxM* bacteria at 37 °C, which synthesize pentaacylated lipid A. However, at 30 °C, Δ(*clsA lpxM*) could be obtained, although such a derivative exhibited small colony morphology and slow-growth phenotype. These results suggest that the transport of tetraacylated and pentaacylated lipid A species could be aided by cardiolipin. The observed essentiality of ClsA in the absence of LpxL, along with the conditional synthetic lethality of Δ(*clsA lpxM*) bacteria, suggest that underacylated lipid A requires cardiolipin for its transport, in addition to the higher demand for MsbA. Consistent with a role for ClsA in participating in the translocation of underacylated LPS, several suppressor mutations that overcome the lethality of Δ(*clsA lpxM*) bacteria can be mapped to the *msbA* gene, strongly supporting the coordinated participation of MsbA and ClsA in LPS trafficking. The majority of such single-amino-acid substitutions are located in domains shown in structural studies to map to either the LPS-binding domain, or the periplasmic exit surface groove of MsbA predicted to be in lipid A export pathway [44,45,46]. This could specifically be the case for the Met160 and Ser164 residues of MsbA, and their substitutions could relax the export of underacylated species of lipid A. Furthermore, substitutions like MsbA498D to Y and MsbA529N to K are predicted to cause an expansion of the MsbA NBD cavity, and could lead to enhanced stimulation of ATPase activity, allowing for the transport of tetra- and pentaacylated LPS. Interestingly, MsbA498V mutation was already identified as a suppressor of Δ(*lpxLPM*) and Δ(*waaC lpxMLP*) bacteria, which synthesize only tetraacylated LPS, resulting in restoration of growth at 37 °C in rich media [19]. However, further biochemical studies will be required in order to address how ClsA directly participates in lipid A trafficking. At the same time, an indirect effect leading to the above-mentioned synthetic lethality cannot be ruled out, as Δ*clsA* mutants have pleiotropic phenotypes affecting the protein secretion system and cell division, as well as preventing the DksA-mediated suppression of strains lacking peptidyl–prolyl *cis*/*trans* isomerases [43,61,62]

Aside from identifying factors that limit the growth of strains lacking WaaA Kdo transferase, we also sought to understand the molecular basis of the severe growth defects in strains lacking WaaC heptosyltransferase I and phosphoethanolamine transferase EptB. The LPS of Δ*waaC* strains is composed of Kdo_2_-LA_hexa_, and can incorporate *P-EtN* either on lipid A or on the second Kdo, depending upon the genetic background [19]. However, when EptB and EptA are intact, the Δ*waaC* and Δ*waaF* strains predominantly incorporate *P-EtN* on the second Kdo, at the expense of *P-EtN* incorporation in the lipid A part [19,29,33]. This has been explained as being due to the induction of RpoE-dependent transcription of *eptB* in the Δ*waaC* and Δ*waaF* strains [29]. The expression of *eptB* mRNA is also subjected to negative regulation by the PhoP/Q-regulated MgrR sRNA [31,32]. Upon PhoP/Q induction, MgrR translationally represses the *eptB* mRNA [32]. However, this repression is overcome when the RpoE is induced, as is the case in Δ*waaC* and Δ*waaF* backgrounds [29]. Thus, we further probed the physiological significance of *P-EtN* modification on the second Kdo in Δ*waaC* backgrounds. We took the advantage of the previously reported Ca^++^ sensitivity of Δ(*waaC eptB*) bacteria [19,30,31], and isolated suppressors that could overcome this sensitivity. Suppressor mutations that overcome the Ca^++^ sensitivity of Δ(*waaC eptB*) bacteria mapped to the *basS* and *basR* genes. BasS/BasR constitutes a two-component system (TCS), whose induction leads to the incorporation of non-stoichiometric lipid A modification by *P-EtN* and L-ArnT-dependent Ara4N [28,48]. In this TCS, BasS acts as a sensory histidine kinase, while BasR acts as a response regulator, and together they induce transcription of the *eptA* and *arn* genes [19,48,63]. Thus, two single-amino-acid exchanges—BasS A20D and BasR G53V—were found to confer Ca^++^ resistance, and this was shown to be due to the constitutive induction of the *eptA* and *arn* genes. This was supported by transcriptional analysis using *eptA*–*lacZ* and *arnB*–*lacZ* fusions, as well asd by mass spectrometric analysis of the LPS of Δ(*waaC eptB*) derivatives, with or without the presence of suppressor mutations. Thus, the LPS of the Δ(*waaC eptB*) *basS* A20D and Δ(*waaC eptB*) *basR* G53V strains were found to carry lipid A substitutions with *P-EtN,* as opposed to its absence in the parental Δ(*waaC eptB*) strain under identical growth conditions that do not cause the induction of the *basS/R* regulon. Thus, the Ca^++^ sensitivity of Δ(*waaC eptB*) can be ascribed to the lack of *P-EtN* on the second Kdo, and can be compensated for if transcription of the *eptA* gene is constitutively induced, causing the incorporation of *P-EtN* in the lipid A. Consistent with such a model, we could show that overexpression of the *eptA* gene from a plasmid can also overcome the Ca^++^ sensitivity phenotype of Δ(*waaC eptB*) bacteria. Thus, these results identify the previously unknown mechanism of Ca^++^ sensitivity of Δ(*waaC eptB*) bacteria, along with the importance of *P-EtN* incorporation either in lipid A or on the second Kdo. In this scenario, it is also important to highlight that Ca^++^ sensitivity in the absence of EptB is only observed when either *waaC* or *waaF* are absent, but not in strains with Δ(*waaP eptB*) and Δ(*waaG eptB*) combinations. These results gain support from our earlier observations that the Δ*waaG* and Δ*waaP* strains exhibit *P-EtN* incorporation on the lipid A as well as on the second Kdo—unlike the Δ*waaC* or Δ*waaF* strains, which uniquely show the primary incorporation of *P-EtN* on the second Kdo due to the higher level of induction of the *eptB* gene, whose transcription is induced by the RpoE-dependent envelope stress response [19,29].

Another important observation from this study is the absence of *P-EtN*—as revealed by mass spectrometric analysis of the lipid A part of the Δ*mgrR* strain—compared with *P-EtN*’s presence in the spectra of the wild-type strain under growth conditions that induce the incorporation of lipid A modifications (phosphate-limiting minimal media). This can be best explained on the basis of the lack of translation repression in the absence of MgrR [32]. Thus, Δ*mgrR* bacteria would preferably incorporate *P-EtN* on the second Kdo and, due to competition for the same precursor, phosphoethanolamine is absent in the lipid A part of the LPS in such bacteria. These results also imply that *P-EtN* incorporation on the second Kdo is preferable to its presence in the lipid A. Thus, physiologically, *P-EtN* substitution on the second Kdo confers some growth advantages, as well as leading to switching in the synthesis of glycoform IV, with Rha shifted to the third Kdo instead of attachment to the second Kdo [33].

To further address the molecular basis of defects associated with the absence of WaaC heptosyltransferase I, we further elaborated on the synthetic lethality observed with a Δ(*waaC surA*) combination [19]. SurA is a major periplasmic folding factor [64,65,66]. SurA is known to be involved in the maturation and transport of *β*-barrel OMPs [64,65,67,68,69]. Thus, Δ*surA* bacteria contain fewer mature *β*-barrel OMPs compared to the wild-type strain [64,65,66]. Consequently, Δ*surA* bacteria exhibit hyperinduction of the RpoE-dependent envelope stress response due to defects in OMP folding. LPS core truncations, as observed in strains lacking GmhD or WaaC, also exhibit an elevated induction of RpoE regulon and reduction in OMPs [19,64]. This can partly explain the synthetic lethality of a Δ(*waaC surA*) combination. Here, to extend this work, we found that a gain-of-function mutation in OppA can allow for the growth of Δ(*waaC surA*) bacteria, although such bacteria still exhibit the deep-rough phenotype. Modeling of OppA S273G suppressor mutation on the available crystal structure suggests that the substrate-binding cavity could be expanded, which could alter the recognition of its substrates. OppA is a major periplasmic oligopeptide-binding protein, and a chaperone-like function has been ascribed to it [52,53]. However, more work will be needed in order to understand how OppA S273G can change the protein-folding landscape in the periplasm of *E. coli*. We observed that neither excess nor reduction of OppA can overcome the synthetic lethality of Δ(*waaC surA*) combination, based on genetic experiments presented in this work. We also noted that OppA quantities are unchanged in freshly transduced Δ*waaC* bacteria compared to the wild-type strain. Recently observed elevated levels of OppA unique to Δ*waaC* can be best explained by the usage of untransduced derivatives [70].

Finally, in this work, we addressed additional levels of control of the first committed step in LPS biosynthesis, catalyzed by the essential enzyme LpxC. The in vivo amounts of LpxC are tightly regulated, yet all aspects of the regulation of LpxC quantities other than proteolytic control by LapB/LapC/FtsH complex and by protease HslUV are unknown [10,12,13]. In this work, we identified that one of the most conserved and abundant sRNA—GcvB—negatively controls LpxC synthesis. This was supported by the reduction in LpxC quantities upon overexpression of GcvB sRNA, a partial suppression of the lethality of Δ*lapAB* bacteria, and the sensitivity of Δ*gcvB* strains to the LpxC inhibitor CHIR090. The observed sensitivity of a strain lacking GcvB to CHIR090 strongly supports a role for this sRNA in regulating LpxC synthesis, as such a phenotype is not obvious for the deletion derivatives of other tested sRNA-lacking strains. In the absence of LapB, LpxC is stabilized, causing increased synthesis of LPS [9,71]. Surprisingly, only a minor reduction in LpxC accumulation was observed when GcvB was overexpressed in *lapAB* mutants; thus, the additional level of control of LpxC synthesis by GcvB is possible. Therefore, whether or not GcvB directly inhibits *lpxC* mRNA translation via the base-pairing mechanism needs to be addressed in future. GcvB controls the expression of more than 1% of mRNAs [36,37,38], and could also exercise such control in an indirect manner.

Thus, in summary, this work describes the first construction of suppressor-free strains that lack the entire *waa* locus and are shown to synthesize LPS composed of lipid IV_A_ precursor, with limited growth at 21 °C. This lipid IV_A_ precursor is shown to act as an acceptor for acylation by the LpxL, LpxM, and LpxP enzymes under slow- growth conditions, generating glycosylation-free pentaacylated and hexaacylated species. Suppressors that allow for the growth of such bacteria mapped to the predicted lipid A-binding domain of MsbA, which facilitates LPS exit, and also to the domain that provides a selective function for MsbA in the selection of the acyl chain length of lipid A. This work also discovered a previously unknown requirement of cardiolipin biosynthesis in strains lacking either myristoyl or lauroyl acyl chains, as shown by the synthetic lethality of (*clsA lpxM*) and (*clsA lpxL*) deletion combinations. Finally, we address the importance of EptB in the absence of WaaC with regard to permeability defects that manifest in sensitivity to Ca^++^, and address the potential translational regulation of LpxC by the GcvB sRNA. The Δ(*gmhD*–*waaA*) strains described in this work should allow for the efficient glycoengineering of therapeutic proteins as a source for the production of endotoxin-free substances and further examination of the in vivo and in vitro trafficking of LPS by MsbA. At the time of submission of this manuscript, an independent study has also shown synthetic lethality of Δ(*clsA lpxM*) bacteria. However, in that work, the function of ClsA in deletion derivatives of *waaA*, *lpxL*, *lpxP*, (*lpxLMP*), and *gmhD*-*waaA*—as well as the viability of Δ(*clsA lpxM*) at 30 °C—has not been addressed [72].

## 4. Materials and Methods

### 4.1. Bacterial Strains, Plasmids and Media

The bacterial strains and plasmids used in this study are described in Table 1. Luria–Bertani agar (LA) or—for liquid culture—broth (LB) (Difco, Franklin Lakes, NJ, USA), M9 minimal media (Difco), and phosphate-limiting minimal media were prepared, as described earlier [9,19]. When required, the media were supplemented with ampicillin (100 μg/mL), kanamycin (50 μg/mL), tetracycline (10 μg/mL), chloramphenicol (20 μg/mL), and spectinomycin (50 μg/mL). Polymyxin B (0.25 or 1 μg/mL) or CHIR090 (0.004 or 0.008 μg/mL) were added to LA media when required. The deletion derivatives used in this study of *waaA*, *waaC*, *waaF*, *eptB*, *mgrR*, Δ(*lpxL lpxM lpxP*), Δ(*eptB waaC*), and Δ(*lapAB*) have been previously described [9,19,29,33]. The Δ*gcvB* strain was constructed via the replacement of the coding region by *ada* cassette using λ-Red-mediated recombineering [42]. Similarly, a Δ*clsA* strain was constructed via the replacement of the coding region by *aph* cassette from pKD13 using λ-Red-mediated recombineering [42]. To generate the deletion of the entire *waa* locus, appropriate oligonucleotides were used to amplify antibiotic cassettes, using pKD3 and pKD13 as templates [42]. For PCR reactions, 70-mer forward oligonucleotides containing 50 nt from either the *gmhD* or the *waaF* upstream of the ATG codon and the reverse oligonucleotides, were the same as described for the construction of the Δ*waaA* deletion [19]. PCR products from such amplification reactions were electroporated into BW25113-derivative GK1942 containing the λ-Red recombinase-encoding plasmid pKD46 [19,42]. Transformants were plated in M9 minimal medium at 21 °C and incubated for 72–96 h. To ensure the absence of any extragenic suppressors, bacteriophage T4 lysates were grown in cultures obtained from transformations of Δ(*gmhD–waaA*) and Δ(*waaF–waaA*) and used to transduce into either W3110 or BW25113 wild-type strains. To construct the Δ(*gmhD–waaA lpxL lpxM lpxP*) strain, a previously well-characterized strain—SR7781 Δ(*lpxL lpxM lpxP*)—served as a recipient in bacteriophage T4-mediated transductions, using lysate grown on a Δ(*gmhD–waaA*) strain. Each deletion was verified by PCR amplification using the appropriate chromosomal DNA as a template, sequencing of PCR products, and complementation analysis.

In order to construct single-copy chromosomal *oppA*::3xFLAG, FLAG epitope was added at the C-terminal end of the *oppA* gene, using PCR with pSUB11 [73] serving as a template with appropriate oligonucleotides. PCR products were electroporated into GK1942 using λ-Red recombinase-encoding plasmid pKD46. The presence of 3xFLAG was verified by PCR and linkage with the previously described *clsA:*:Tn*10* mutation [43]. The *oppA*::3xFLAG was further introduced into the wild-type strain and its Δ*waaC* derivative via bacteriophage P1-mediated transductions. Subsequently, the *aph* cassette was excised by the introduction of Ts FLP recombinase plasmid pCP20, followed by the plasmid’s removal by culturing antibiotic-free derivatives at 37 °C.

### 4.2. The Isolation of Extragenic Suppressors of Δ(gmhD-waaA) Mutants and Their Mapping

As suppressor-free Δ(*gmhD–waaA*) bacteria exhibit restricted growth in the M9 minimal medium at 21 °C, several independent cultures of each strain were grown at 21 °C, and portions of each were plated at 32 °C in LB medium in order to isolate suppressors. After verification of their growth in LB medium at 32 °C, mutations were marked with mini-Tn*10* Kan, as described previously [12,74], and verified that the Tn*10*-linked suppressor mutation breeds true by backcrossing into Δ(*gmhD–waaA*) strains. In order to identify the gene in which the suppressor mutation exists, the position of Tn*10* was obtained by first recombining Tn*10* onto a previously described cosmid library [75], and was used for subcloning. The position of Tn*10* was obtained via inverse PCR using the chromosomal DNA of a suppressor-containing strain as a template [12]. All Tn*10*-recombinant cosmids were found to carry the *msbA* gene. As the Tn*10* was found to be linked to the *msbA* gene, the *msbA* and downstream *lpxK* genes were PCR-amplified, and the PCR products were subjected to DNA sequencing. In order to move the suppressor mutation in the *msbA* gene into other strains, a Kan cassette was inserted into the *ycaI* gene located upstream of the *msbA* gene, and suppressor alleles of *msbA* were moved out via T4-mediated transductions. The presence of *msbA* mutation was verified by PCR and linkage with the *ycaI* gene and with the tightly linked *cmk* gene [43]. The same strategy was employed in order to identify temperature-resistant suppressors of Δ(*lpxM clsA*) bacteria. Cultures of a Δ(*clsA–lpxM*) strain were grown under permissive growth conditions of 30 °C and plated at 43 °C in LA medium. Marking of suppressor mutations and their mapping, followed by sequencing of chromosomal DNA using *msbA*-specific oligonucleotides, was carried out as described above.

### 4.3. The Isolation of Ca^++^-Resistant Extragenic Suppressors of Δ(waaC eptB) Mutants and Their Mapping

Since Δ(*waaC eptB*) strains exhibit extreme sensitivity to supplementation with non-lethal concentrations of CaCl_2_, extragenic suppressors were sought. Thus, cultures of Δ(*waaC eptB*) bacteria were plated in LA medium supplemented with either 5 mM or 7 mM of CaCl_2_. Ca^++^-resistant colonies were streak-purified and verified for their phenotype. Next, 12 independent Ca^++^-resistant Δ(*waaC eptB*) derivatives were used for the marking of the suppressor mutation by Tn*10*. Nine of them were found to have a single mutation linked to the Tn*10*. The position of the Tn*10* was determined as described in Section 4.2, using DNA products obtained via inverse PCR. To identify the exact mutation, *eptA*, *basS*, *basR*, *pmrR*, and the adjacent chromosomal DNA were PCR-amplified and subjected to DNA sequencing. For the quantification of sensitivity to CaCl_2_, isogenic cultures were grown in LB medium and tested via spot-dilution assay in LA supplemented with 2.5–7 mM of CaCl_2_.

### 4.4. Quantification of BasS/R Activation by the Measurement of eptA–lacZ and arnB–lacZ Activity

To measure the activity of BasS/R-regulated promoters, two well-characterized reporter systems were used by studying the promoter activities of the *eptA* and *arnB* genes. Construction of single-copy chromosomal *eptA*–*lacZ* and *arnB*–*lacZ* promoter fusion has been previously described [19]. For the measurement of promoter activities, *basS* A20D and *basR* G53V mutations were transduced into strains carrying a single-copy chromosomal promoter fusion of *eptA*–*lacZ* and *arnB*–*lacZ*, either with intact *eptB* and *waaC* genes, or in the background of the Δ(*waaC eptB*) mutation. For the *β*-galactosidase assay, isogenic bacterial strains were grown in LB medium at 37 °C until their exponential growth phase and then harvested by centrifugation. Bacterial pellets were resuspended in LB medium and adjusted to an OD_595_ of 0.03. Cultures were allowed to grow aerobically for another 90 min and aliquots were drawn at different growth intervals. The *β*-galactosidase activity was measured in Miller units, as previously described [76]. At least three independent cultures were assayed for each isogenic strain.

### 4.5. Cloning, Expression and Disruption of GcvB sRNA

In order to clone the *gcvB* gene, the minimal coding sequence was assembled via Gibson cloning and cloned behind the *lac* promoter in the modified pBR322 vector [43]. The *gcvB* gene was cloned using the EcoRI and BamHI restriction endonuclease sites, and when required the expression was induced by the addition of IPTG at the concentrations described in the Results section.

### 4.6. Western Blot Analysis and Immunodetection of OppA::3XFLAG and LpxC

Thirty milliliter isogenic bacterial cultures, carrying chromosomal *oppA* with 3xFLAG appended at the C-terminal end, were grown at 37 °C in LB medium until their exponential growth phase. Similarly, isogenic bacterial strains carrying either the vector alone or the plasmid expressing GcvB sRNA were grown in LB medium with appropriate antibiotics. The expression of *gcvB* sRNA was induced by the addition of varying concentrations of IPTG, as indicated. Cultures were harvested by centrifugation at 7000 rpm for 15 min and resuspended in SDS lysis buffer. Equivalent amounts of proteins were applied to a 12.5% SDS–PAGE, and after electrophoresis, proteins were blotted to a PVDF membrane. Blots were probed with the anti-FLAG-specific M2 monoclonal antibody (F3165 from Sigma) for the detection of OppA-FLAG. For the detection of LpxC, blots were probed with the anti-LpxC polyclonal antibody, as described previously [9,12]. Protein intensity in the blots was revealed using a chemiluminescence kit from Thermo Scientific (Warsaw, Poland), as per the manufacturer’s instructions.

### 4.7. LPS Extraction and Its Analysis

Typically, 400 mL of bacterial cultures were grown in a rotary shaker at 250 rpm in LB medium for Δ(*waaC eptB*) derivatives and for the analysis of Δ*mgrR* and its parental wild type in phosphate-limiting medium at 37 °C until an OD_595_ of 0.8–1.00. Cultures were harvested by centrifugation at 7000 rpm for 30 min, and the pellets were dried. LPS was extracted using the phenol/chloroform/petroleum ether procedure [77]. For the LPS analysis, lyophilized material was dispersed in water by sonication and then resuspended at a concentration of 2 mg/mL [19]. For suppressor-free strains lacking WaaA Kdo transferase, and their derivatives lacking the *waa* locus, with or without concomitant deletion of (*lpxL lpxM lpxP*) genes, cultures were grown in M9 minimal medium at 21 °C up to an OD_595_ of 0.6. When needed, cultures grown in M9 minimal medium at 21 °C were harvested by centrifugation and then resuspended in LB medium either at 21 °C or at 30 °C, at an OD_595_ of 0.2, and then allowed to grow for another 6 h prior to harvesting by centrifugation. Cultures of Δ(*gmhD-waaA*) derivatives with suppressor mutation in the *msbA* gene were grown in LB medium at 37 °C until an OD_595_ of 0.6 with appropriate antibiotics. LPS of strains synthesizing lipid IV_A_ and its derivatives were extracted, following the procedure used for the isolation of deep-rough LPS [19,78]. Glycerophospholipids and lipid A mixture were resuspended in a chloroform/methanol mixture (4:1, *v*/*v*) at a concentration of 2 mg/mL. To validate the absence of Kdo, LPS from Δ(*gmhD–waaA*) derivatives were subjected to TLC immunostaining using the A20 or A6 monoclonal antibodies [57,79,80], with the necessary controls as described previously [19]. Phospholipid composition was analyzed by GLC–MS analysis and by thin-layer chromatography.

### 4.8. Mass Spectrometry

Electrospray ionization Fourier-transform ion cyclotron (ESI FT–ICR) mass spectrometry was performed on either intact LPS or using glycerophospholipids and lipid A mixture in the negative ion mode. LPS samples were dissolved at a concentration of ∼10 ng/μL and analyzed, as previously described [33]. For the acquisition of mass spectra, an APEX QE (Bruker Daltonics, Bremen, Germany) equipped with a 7-tesla actively shielded magnet and dual ESI-MALDI was used. Mass spectra were charge-deconvoluted, and the mass numbers given refer to the monoisotopic peaks. Mass calibration was done externally using well-characterized compounds of known structures, as described previously [19,81].

## Figures and Tables

**Table 1:** Strains used in this study. Additional strains are shown in Section 2.6.

Strains	Genotype	Reference
BW25113	*lacI*^q^ *rrnB*_T14_ Δ*lacZ*_WJ16_ *hsdR514* Δ*araBAD*_AH33_ Δ*rhaBAD*_LD78_	[42]
W3110	*λ*^-^ *IN*(*rrnD-rrnE*)*1 rph-1*	Our collection
GK1111	W3110 Δ*lac*	[19]
SR9900	W3110 Δ(*waaF*–*waaA*)	This study
SR9902	W3110 Δ(*gmhD*–*waaA*)	This study
SR9919	SR9902 *msbA* P50S	This study
SR10123	SR9902 *msbA* R310S	This study
SR7781	W3110 Δ(*lpxL lpxM lpxP*)	[13]
SR23225	SR7781 Δ*waaA*	This study
SR23234	SR7781 Δ(*gmhD*–*waaA*)	This study
GK1162	W3110 *eptB*<>*aph*	[19]
GK1181	W3110 *eptB<>frt*	[33]
SR15676	GK1181 *waaC*<>*aph*	This study
SR8649	GK1181 *waaC*<>*cat*	This study
SR8503	SR8649 *basS* A20D	This study
SR23091	SR8503 Δ*lac*	This study
SR23111	SR23091 φ(*eptA*–*lacZ*)	This study
SR23114	SR23091 φ(*arnB*–*lacZ*)	This study
SR23035	GK1111 *basR* G53V	This study
GK6128	SR23035 φ(*eptA*–*lacZ*)	This study
SR23061	SR23035 *eptB*<>*aph*	This study
SR23065	SR23061 *waaC*<>*cat*	This study
SR19143	GK1111 φ(*arnB*–*lacZ*)	This study
SR23075	SR19143 *basR* G53V	This study
GK2048	BW25113 *waaF*<>*cat*	[29]
SR23201	GK1181 *waaF*<>*cat*	This study
SR23205	GK1181 *waaG*	This study
SR23212	GK1181 *waaP*	This study
GK2576	W3110 *mgrR*<>*ada*	This study
SR17187	BW25113 *lapA–lapB*<>*aph*	[9]
SR20491	BW25113 *gcvB*<>*ada*	This study
SR22995	SR20491 Δ(*lapA lapB*)	This study
SR22990	BW25113 p(*plac*–*gcvB*^+^)	This study
SR8320	W3110 Δ(*surA waaC*)	This study
SR23138	W3110 *oppA*<>*ada*	This study
SR23163	W3110 *oppA*::3XFLAG	This study
SR23179	SR23163 *waaC*<>*cat*	This study
GK5504	BW25113 *clsA*::Tn*10*	[43]
SR23279	SR15676 p*eptA*^+^	This study

**Table 2:** Determination of the viability of Δ*waaA* derivatives in the wild type, Δ(*lpxL lpxM lpxP*) and Δ*ftsH* strains by bacteriophage-mediated transductions. ND: not determined; sc: small colonies. Numbers indicate the number of transductants using an equivalent amount of recipient.

Number of Transductants					
Recipient	Donor				
	Δ*waaA*		Δ(*gmhD*–*waaA*)		Δ*ftsH sfhC21*
	M9 21 °C	LA 21 °C	M9 21 °C	LA 21 °C	M9 21 °C
wild type	3912	430 sc	3211	390 sc	0
Δ(*lpxL lpxM lpxP*)	3890	0	3374	0	ND
Δ*waaA* + vector alone	ND		ND		750
Δ*waaA* + p*ftsH*^+^	ND		ND		966

**Table 3:** Suppressor mutations that rescue the conditional lethality of Δ(*lpxM clsA*) mapping to the *msbA* gene.

Strains	Mutation Position	Amounts of Isolates
SR23302 SR23318 SR23319	S120L (TCA→TTA)	3
SR23305 SR23307	M160I (ATG→ATA)	2
SR23315	S164C (AGT→TGT)	1
SR23313	V287A (GTT→GCT)	1
SR23309 SR23317	D431Y (GAT→TAT)	2
SR23316	D498Y (GAT→TAT)	1
SR23303	I177M (ATT→ATG) N529K (AAC→AAA)	1

**Table 4:** Single amino exchange in the *oppA* coding sequence S273G allows for the construction of Δ(*waaC eptB*) deletion. Determination of the viability of Δ(*waaC* *surA*) derivatives in the presence or absence of *oppA* S273G suppressor mutation by bacteriophage-mediated transductions. Numbers depict the number of transductants using the equivalent amount of recipient.

Number of Transductants				
Recipient	Donor			
	Δ*surA*			
	M9 30 °C	LA 30 °C	M9 37 °C	LA 37 °C
Δ*waaC*	3	6	4	5
Δ*waaC oppA* S273G	1034	1190	1274	960

**Table 5:** Overexpression of GcvB suppresses lethality of a Δ(*lapA lapB*) derivative. Determination of the viability of Δ(*lapA lapB*) derivatives in the presence of the vector alone or when the expression of either *gcvB* sRNA or *slrA* sRNA is induced. Numbers indicate the number of transductants using the equivalent amount of recipient. ND: not determined.

Number of Transductants				
Recipient	Donor			
	Δ*lapAB*		*lppA*::Tn*10*	
	M9 30 °C	LA 37 °C	M9 30 °C	LA 37 °C
wild type + vector alone	530	5	3830	3450
wild type + p*gcvB*^+^ + IPTG 90 μM	3102	2545	ND	ND
Δ*lapAB* + vector alone	ND	ND	2574	2956
Δ*lapAB* + p*slrA*^+^ + IPTG 90 μM	2975	3343	ND	ND

## Data Availability

All data are contained within the article.
